# The CRTC1-MAML2 fusion is the major oncogenic driver in mucoepidermoid carcinoma

**DOI:** 10.1172/jci.insight.139497

**Published:** 2021-04-08

**Authors:** Zirong Chen, Wei Ni, Jian-Liang Li, Shuibin Lin, Xin Zhou, Yuping Sun, Jennifer W. Li, Marino E. Leon, Maria D. Hurtado, Sergei Zolotukhin, Chen Liu, Jianrong Lu, James D. Griffin, Frederic J. Kaye, Lizi Wu

**Affiliations:** 1 Department of Molecular Genetics and Microbiology,; 2 UF Health Cancer Center, and; 3 Genetics & Genomics Graduate Program, UF Genetics Institute, University of Florida College of Medicine, Gainesville, Florida, USA.; 4 Integrative Bioinformatics, National Institute of Environmental Health Sciences, Research Triangle Park, North Carolina, USA.; 5 Department of Pathology, Immunology and Laboratory Medicine,; 6 Department of Biochemistry and Molecular Biology, and; 7 Department of Medicine, University of Florida College of Medicine, Gainesville, Florida, USA.; 8 Division of Endocrinology, Diabetes, and Nutrition, Department of Medicine, Mayo Clinic Health System La Crosse, Wisconsin, USA, and; 9 Mayo Clinic, Rochester, Minnesota, USA.; 10 Department of Pediatrics, University of Florida College of Medicine, Gainesville, Florida, USA.; 11 Department of Pathology, Yale School of Medicine, Yale New Haven Hospital, New Haven, Connecticut, USA.; 12 Department of Medical Oncology, Dana-Farber Cancer Institute, Boston, Massachusetts, USA.

**Keywords:** Oncology, Head and neck cancer, Mouse models, Oncogenes

## Abstract

No effective systemic treatment is available for patients with unresectable, recurrent, or metastatic mucoepidermoid carcinoma (MEC), the most common salivary gland malignancy. MEC is frequently associated with a t(11;19)(q14-21;p12-13) translocation that creates a *CRTC1-MAML2* fusion gene. The CRTC1-MAML2 fusion exhibited transforming activity in vitro; however, whether it serves as an oncogenic driver for MEC establishment and maintenance in vivo remains unknown. Here, we show that doxycycline-induced CRTC1-MAML2 knockdown blocked the growth of established MEC xenografts, validating CRTC1-MAML2 as a therapeutic target. We further generated a conditional transgenic mouse model and observed that Cre-induced CRTC1-MAML2 expression caused 100% penetrant formation of salivary gland tumors resembling histological and molecular characteristics of human MEC. Molecular analysis of MEC tumors revealed altered p16-CDK4/6-RB pathway activity as a potential cooperating event in promoting CRTC1-MAML2–induced tumorigenesis. Cotargeting of aberrant p16-CDK4/6-RB signaling and CRTC1-MAML2 fusion–activated AREG/EGFR signaling with the respective CDK4/6 inhibitor Palbociclib and EGFR inhibitor Erlotinib produced enhanced antitumor responses in vitro and in vivo. Collectively, this study provides direct evidence for CRTC1-MAML2 as a key driver for MEC development and maintenance and identifies a potentially novel combination therapy with FDA-approved EGFR and CDK4/6 inhibitors as a potential viable strategy for patients with MEC.

## Introduction

Fusion cancer genes are unique diagnostic and therapeutic biomarkers due to their selective expression in specific tumor subtypes and their detection as core somatic mutational events that predict an early role in initiation of tumorigenesis (1–3). For example, the successful clinical development of targeted therapies for fusion-associated cancers, including BCR-ABL–positive chronic myeloid leukemia, ALK fusion–positive lung cancers, and NTRK fusion–positive solid tumors, emphasizes the importance of understanding fundamental mechanisms of oncogenic fusion genes and has revolutionized clinical management (4–6). However, there remain unmet needs for other cancer subtypes harboring signature chromosomal translocations. Defining the mechanisms that underlie tumorigenesis in these cases will provide new treatment opportunities and may offer unexpected insights into cancer signaling pathways that have potential broad implications for cancer biology.

Mucoepidermoid carcinoma (MEC) is the most common subtype of salivary gland malignancies with distinct histological features (7–9). MEC tumors display cellular heterogeneity containing various proportions of mucin-secreting cells, epidermoid cells, and cells of intermediate type with varying architectural formations from cystic structures to small solid nests or glandular-like structures. MEC tumors can also arise infrequently from other sites such as the lung, pancreas, cervix, and breast (10–14). The only effective management of MEC is surgical resection with postoperative radiation in selected cases. However, high-grade and occasionally cases of intermediate- and low-grade MEC tumors can subsequently develop unresectable or metastatic disease with lethal outcome due to lack of effective systemic treatment. A recurrent t(11;19)(q14-21;p12-13) translocation encoding a potentially novel *CRTC1-MAML2* gene fusion has been detected in up to 80% of MEC cases in studies of multiple cohorts (15–18). CRTC1-MAML2 was also detected in a subset of Warthin’s tumors, the second most common benign salivary gland tumors; however, in retrospect, these tumors invariably contained small foci of MEC (19, 20). There are 3 distinct CRTC gene family members in mammalian species (21). More recently, a CRTC3-MAML2 fusion variant was detected in up to 6% of MEC cases and was mutually exclusive to detection of CRTC1-MAML2 (15, 22). Therefore, CRTC1/3-MAML2 fusion is highly specific for MEC. The t(11;19) translocation is occasionally the sole cytogenetic alteration in MEC salivary gland tumors (23) and is also detected in nonsalivary gland MEC–like tumors throughout the body (8, 10), suggesting that the acquired CRTC1-MAML2 fusion is an early core event in MEC pathogenesis. Therefore, a comprehensive understanding of mechanisms of CRTC1-MAML2–mediated tumorigenesis and requirements for sequential cooperating somatic events will be critical for development of effective treatments.

We initially cloned the *CRTC1-MAML2* fusion gene from human MEC–derived cell lines carrying a recurrent t(11;19) translocation using positional cloning (24). *CRTC1* (also known as *MECT1*, *TORC1*, *WAMPT1*) belongs to the CREB-regulated transcription coactivator (CRTC) family with 3 members (CRTC1–3) that play critical roles in metabolism, aging, memory, and cancer (21), while MAML2 is a member of the mastermind-like coactivator family that is integral for Notch receptor–mediated transcriptional activation and important in development and disease, including cancer (25). The t(11;19) translocation fused exon 1 of the *CRTC1* gene at 19p13 in-frame to exons 2–5 of the *MAML2* gene at 11q21, leading to a chimeric protein composed of the CREB-binding domain (CBD) of CRTC1 (42 aa) at the N-terminus with the transcriptional activation domain (TAD) of MAML2 (983 aa) at the C-terminus (24, 26). The CRTC1-MAML2 fusion potently activated a CREB-dependent transcriptional program with recruitment of p300/CBP into the CREB complex through its MAML2 TAD (26–28). This fusion was also shown to transactivate AP-1 and MYC, likely contributing to CREB-independent activities (29, 30). The CRTC1-MAML2 fusion induced E1A-immortalized rat kidney RK3E epithelial cells to grow into colonies and the transformed RK3E cells formed tumors when s.c. implanted to immunocompromised mice (24, 26, 27, 31). Moreover, shRNA-mediated knockdown of the expression of the CRTC1-MAML2 fusion gene or its target genes such as AREG or LINC00473 impaired the growth and survival of human MEC cells (32, 33). All these data support an oncogenic activity of the CRTC1-MAML2 fusion. However, a definitive role for CRTC1-MAML2 in driving MEC initiation and maintenance in vivo remained unproven. Moreover, the lack of mouse models for MEC impedes our efforts to dissect the tumorigenic mechanisms underlying MEC and explore anti-MEC therapeutics.

In this study, we demonstrate that the CRTC1-MAML2 fusion is an oncogenic driver for MEC establishment and maintenance by generating the first genetically engineered mouse model to our knowledge of CRTC1-MAML2–driven MEC and characterizing a human MEC cell model with inducible CRTC1-MAML2 shRNA expression in vivo. Moreover, we identified altered p16-CDK4/6-RB pathway activity as a potential cooperating event for the CRTC1-MAML2 fusion–induced tumorigenesis and showed that cotargeting of the key driver CRTC1-MAML2–activated AREG/EGFR signaling and the cooperating p16-CDK4/6-RB signaling using FDA-approved EGFR and CDK4/6 inhibitors likely serves as an effective anti-MEC therapeutic strategy.

## Results

### Doxycycline-induced CRTC1-MAML2 knockdown blocks the growth of established MEC tumors.

**Figure 1:**
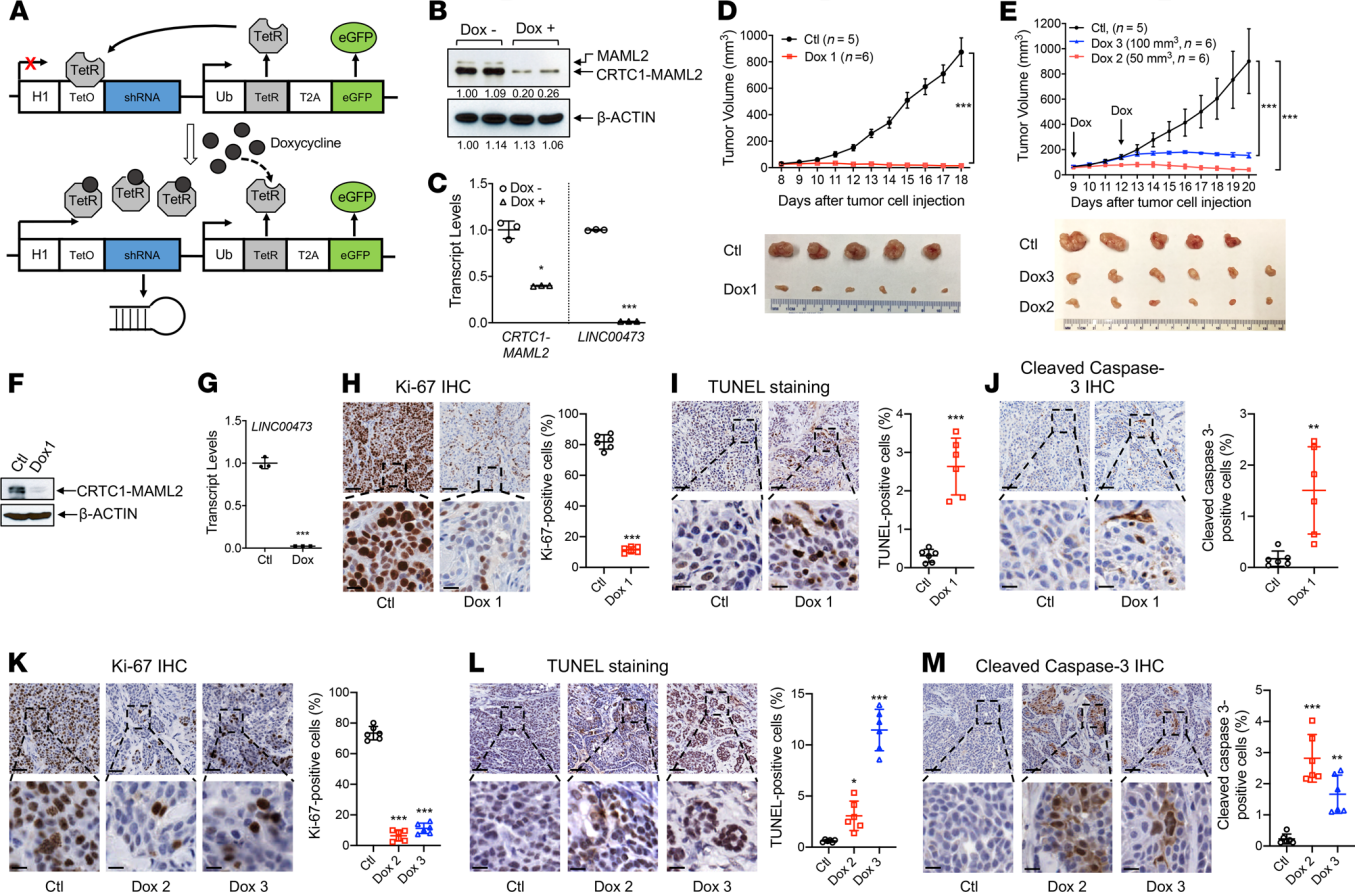
Dox-inducible *CRTC1-MAML2*–targeting shRNAs decreased the growth of established human MEC xenografts by reducing cell proliferation and enhancing apoptosis. (**A**) A diagram of the lentiviral-based vector (FH1tUTG) system for inducible *CRTC1-MAML2* fusion–targeting shRNA expression, adapted from Herold et al. (34). (**B** and **C**) A stable H3118 MEC cell clone with CRTC1-MAML2 ishRNA (H3118-fusion ishRNA) was treated with 1 μg/mL Dox for 72 hours and analyzed for CRTC1-MAML2 fusion knockdown by Western blotting (**B**) and LINC00473 expression by qPCR (**C**). Data are mean ± SD. A 2-tailed *t* test was used to calculate the *P* values (*P < 0.05 and ***P < 0.001). (**D** and **E**) NOD.SCID mice were injected s.c. with 1 × 10^6^ H3118-fusion ishRNA cells per mouse and provided with Dox or control diet starting on the day of tumor cell injection (Dox 1 cohort) (**D**) or when tumors reached approximately 50 mm^3^ (Dox 2 cohort) and approximately 100 mm^3^ (Dox 3 cohort) (**E**). Tumor volumes at various days after tumor cell implantation and the images of resected tumors at the endpoint were shown. (**F** and **G**) The expression levels of the *CRTC1-MAML2* fusion and *LINC00473* in Dox-treated versus control xenograft MEC tumors were analyzed by Western blotting (**F**) and qPCR (**G**), respectively. β-Actin was used as a protein loading control. (**H–M**) Cell proliferation and apoptosis were evaluated on 3 Dox-treated versus control MEC xenograft tumor sections by Ki-67 IHC (**H** and **K**), TUNEL staining (**I** and **L**), and cleaved caspase 3 IHC (**J** and **M**). The positively stained nuclei/cells were quantified in 6 randomly selected visual fields (5×, 1000 × 1000 pixels) using ImageJ. Scale bar: 100 μm (upper panels), 25 μm (lower panels). A 2-tailed *t* test was used for 2-group comparisons and 1-way ANOVA for multiple group comparisons (*P < 0.05, **P < 0.01, ***P < 0.001).

Our previous evidence supported a critical role of the CRTC1-MAML2 fusion in maintaining MEC malignancy, as shown by impaired growth and survival of human MEC cells after constitutive shRNA knockdown of the CRTC1-MAML2 fusion (31, 32). However, these constitutive shRNA knockdown strategies were insufficient to prove an essential role of CRTC1-MAML2 in MEC maintenance in vivo. Therefore, we investigated whether doxycycline-induced (Dox-induced) *CRTC1-MAML2* knockdown blocks the growth of established MEC xenografts. We generated human MEC cell models (H3118-fusion inducible shRNA [ishRNA]) by stably transducing H3118 MEC cells with H1tUTG-based lentiviruses (34) that expressed Dox-inducible CRTC1-MAML2–targeting shRNAs under the control of an H1 promoter (Figure 1A). The shRNA sequences used in this inducible system were previously validated for inducing fusion knockdown–specific effects in human MEC cells (32, 33). A representative H3118 MEC clone showed tight control of shRNA expression with Dox treatment, as evidenced by CRTC1-MAML2 knockdown and inhibition of a validated fusion target gene LINC00473 (33) by Western blotting and quantitative PCR (qPCR) assays (Figure 1, B and C). These H3118-fusion ishRNA cells were s.c. implanted to NOD.SCID mice, which were then randomly divided into 2 groups: one was fed with Dox chow to induce CRTC1-MAML2-shRNA expression, and the other was fed with control diet. Dox chow was provided immediately after tumor cell implantation (Figure 1D) or at the time when the xenografts reached approximately 50 mm^3^ and approximately 100 mm^3^ (Figure 1E). We observed that the Dox-treated groups (with Dox-induced CRTC1-MAML2 shRNA expression) had reduced tumor growth overtime with smaller tumors at the endpoints, as compared with the control groups (Figure 1, D and E). Analysis of the harvested tumors showed that Dox-treated tumors showed a decrease in the expression levels of *CRTC1-MAML2* and *LINC00473* (Figure 1, F and G), a reduction in the number of Ki-67–positive proliferating cells (Figure 1, H and K), and an increase in the number of TUNEL-positive (Figure 1, I and L) and cleaved caspase 3–positive (Figure 1, J and M) apoptotic cells, in comparison with the controls. These data demonstrate that sustained CRTC1-MAML2 expression is required for MEC tumor growth and maintenance in vivo. Therefore, the CRTC1-MAML2 fusion represents a validated therapeutic target for MEC.

### Cre-induced expression of the CRTC1-MAML2 fusion transgene in mouse salivary gland ductal cells results in 100% penetrant development of murine salivary gland MEC tumors.

**Figure 2:**
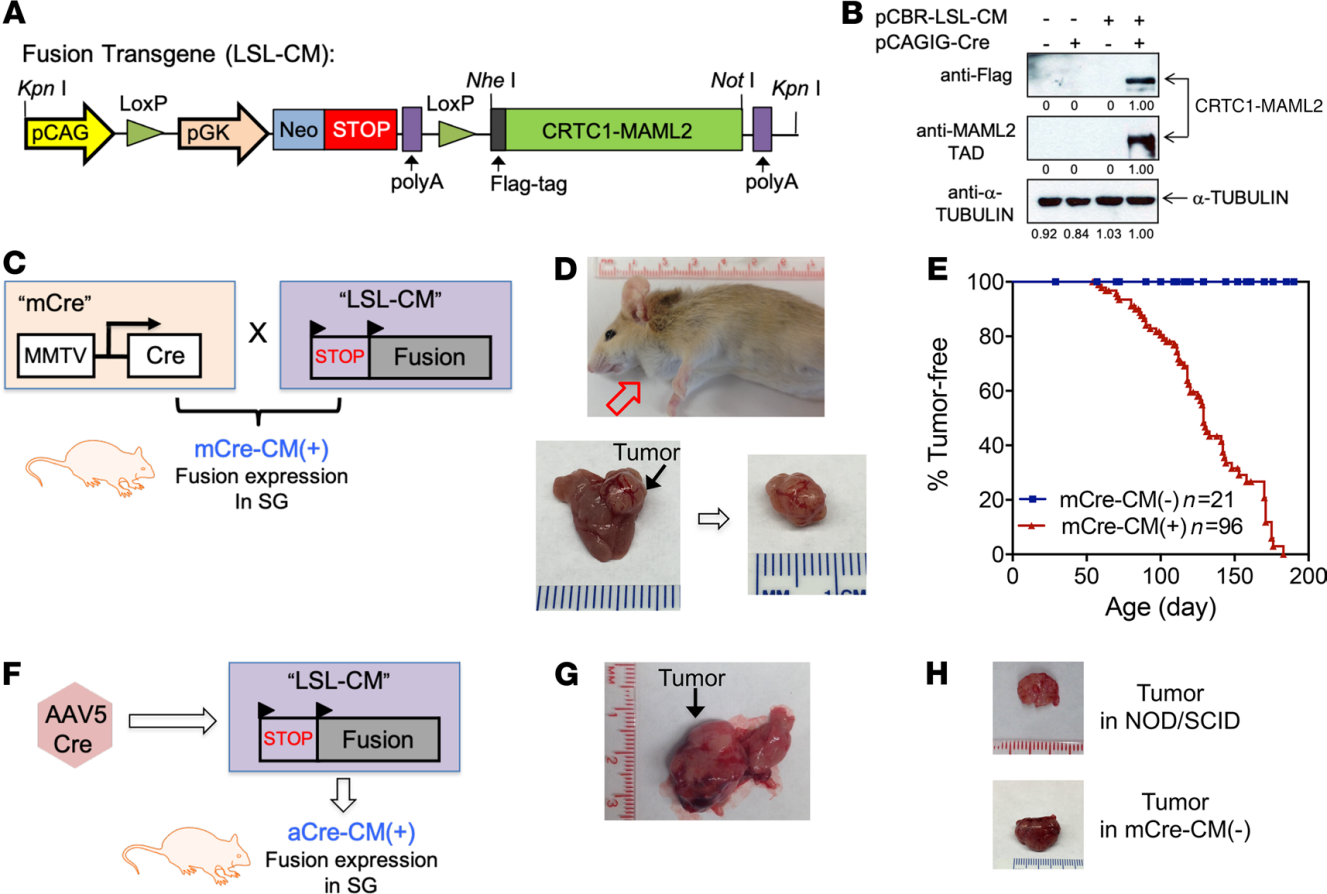
The Cre-regulated *CRTC1-MAML2* transgenic mice developed salivary gland tumors. (**A**) A schematic representation of the Cre/LoxP-mediated *CRTC1-MAML2* fusion transgene construct (LSL-CM). (**B**) Western blotting confirmed Cre-induced expression of CRTC1-MAML2 transgene. The transgenic construct was cotransfected with pCAGIG-Cre plasmid or empty vector into 293T cells, and the CRTC1-MAML2 fusion expression was detected at 36 hours after transfection by Western blotting using anti-Flag antibodies or anti-MAML2 TAD antibodies. α-Tubulin was used as a loading control. (**C**) A schematic diagram shows the crossing of the LSL-CM transgenic mice with MMTV-Cre mice to induce CRTC1-MAML2 transgene expression in salivary glands. The resulting mCre-CM(+) mice were monitored for tumor development. (**D**) A representative mCre-CM(+) mouse developed salivary gland (SG) tumor. (**E**) Salivary gland tumor occurrence was shown in mCre-CM(+) versus nontransgene mCre-CM(–) control cohorts. Half of mCre-CM(+) mice (*n* = 96) developed SG tumors by 129 days, while no tumors were observed in the control mCre-CM(–) mice (*n* = 21). (**F**) A schematic diagram shows ductal delivery of AAV5-Cre-eGFP viruses to salivary glands of LSL-CM(+) mice. The resulting aCre-CM(+) mice were monitored for tumor development. (**G**) A representative aCre-CM(+) mouse developed SG tumor at about 3 months after retrograde injection of AAV5-Cre-eGFP viruses. (**H**) Cell suspensions from primary CRTC1-MAML2–induced salivary gland tumors grew into tumors after s.c. engrafting into immunocompromised NOD.SCID mice or immunocompatible mCre-CM(–) mice containing no *CRTC1-MAML2* transgene.

Although the CRTC1-MAML2 fusion is implicated as an early event in MEC tumorigenesis, direct evidence for the CRTC1-MAML2 fusion in driving MEC initiation remained lacking. To study the in vivo role of the CRTC1-MAML2 fusion in tumorigenesis, we first created a mouse strain carrying the *CRTC1-MAML2*–knock-in allele under control of the *CRTC1* enhancer and promoter to reproduce the genetic abnormality in human MEC (Supplemental Figure 1; supplemental material available online with this article; https://doi.org/10.1172/jci.insight.139497DS1). However, pups carrying the knock-in allele died immediately after birth, preventing further analysis. To circumvent the adverse impact of CRTC1-MAML2 expression on mouse development, we used a strategy that allows conditional expression of CRTC1-MAML2 in a Cre-regulated transgenic mouse model. Here, the transgenic construct consisted of the *CRTC1-MAML2* fusion cDNA downstream of a stop cassette flanked with 2 loxP sites (LoxP-STOP-LoxP-FLAG-tagged CRTC1-MAML2, denoted as LSL-CM) (Figure 2A). The stop cassette can be deleted upon expression of Cre recombinase, leading to FLAG-tagged CRTC1-MAML2 expression under the control of a ubiquitous CMV early enhancer/chicken β actin (CAG) promoter. An N-terminal FLAG tag was previously shown to have no effect on the fusion’s transforming and coactivator functions (24, 26). We first validated Cre-induced expression of the transgenic CRTC1-MAML2 fusion construct in cell culture by Western blotting with anti-FLAG and MAML2 TAD antibodies (Figure 2B) and subsequently isolated the transgenic fragment, LSL-CM, for microinjection into FVB/J zygotes (Supplemental Figure 2A). Three Cre-regulated CRTC1-MAML2 transgenic mouse lines were eventually obtained with an estimated transgene copy number of 15, 4, and 1, respectively (Supplemental Figure 2, B–D).

We next employed 2 approaches of introducing Cre recombinase to induce the *CRTC1-MAML2* transgene expression in mouse salivary glands by using salivary gland–expressing Cre mouse lines and Cre viruses. We first utilized the MMTV-Cre transgenic mouse line (Tg[MMTV-Cre]4Mam/J), as this line expresses Cre in the ductal cells of salivary glands (35), which were postulated to undergo transformation leading to MEC (36, 37). We crossed our 3 Cre-regulated CRTC1-MAML2 transgenic mouse lines (LSL-CM) with homozygous MMTV-Cre transgenic mice (mCre) (Figure 2C). Two cohorts of mice, including bitransgenic mCre-CM(+) mice that carried the *CRTC1-MAML2* transgene and age-matched transgene-negative mCre-CM(–) littermate controls, were obtained and monitored for tumor development. Line 1 (copy number of 15) and Line 2 (copy number of 4), but not Line 3 (copy number of 1), developed tumors. Line 1 consistently developed tumors in the salivary glands (Figure 2D), but Line 2 developed tumors mainly in the flanks and some in the salivary glands (not shown). Salivary gland tumors developed from both of the lines expressed the transgenic CRTC1-MAML2 fusion protein at a level close to that of endogenous fusion in human MEC cells (Supplemental Figure 3). We subsequently focused our characterization mainly on Line 1. These mCre-CM(+) mice developed single palpable salivary gland tumors, starting around 2–3 months of age, and showed 100% tumor penetrance by 6–9 months (median tumor latency time [T50] = 129 days) (Figure 2, D and E). In contrast, no tumors were observed in all the control mCre-CM(–) mice without the fusion transgene (Figure 2E). Western blotting showed that the *CRTC1-MAML2* transgene is expressed in salivary gland tumor and skin with barely detectable expression in multiple tissues examined, including the lung, heart, liver, kidney, intestine, spleen, brain, and muscle (Supplemental Figure 4). Consistent with the fusion expression in the skin, a prominent phenotype outside salivary glands in the mCre-CM(+) mice was the formation of skin cysts, especially around the mouth and ear (Supplemental Figure 5).

We also crossed our transgenic mice with other salivary gland Cre lines to induce transgenic CRTC1-MAML2 expression, including Dcpp-1–CreERT2 (specific for serous demilune cells of the sublingual gland and intercalated duct cells of the parotid gland) and Pip-CreERT2 (specific for acinar cells of submandibular gland) that were developed by Maruyama et al. (38). However, we observed no tumor formation up to 18 months of age (Supplemental Figure 6). Since there appeared low CRTC1-MAML2 expression in salivary glands in these mice by IHC and qPCR assays (Supplemental Figure 6), it remains inconclusive whether these Dcpp-1– or Pip promoter–driven specific cell components can be targeted for CRTC1-MAML2–driven transformation.

Since adeno-associated viruses (AAVs) were previously shown to efficiently transduce cells in salivary glands (39, 40), we delivered AAV5-Cre viruses into the major salivary glandular ducts of the LSL-CM mice by retrograde injection (Figure 2F). The resulting aCre-CM(+) mice developed salivary gland tumors with a latency of approximately 3–4 months (Figure 2G). We further implanted cell suspensions from salivary gland tumors arising from mCre-CM(+) mice s.c. in immunocompromised NOD.SCID mice and immunocompetent mCre-CM(–) mice that contained mCre but not the fusion transgene, and we observed tumor formation around 2 months (Figure 2H), indicating that these CRTC1-MAML2–induced tumor cells were fully transformed. Thus, our combined data demonstrate that the CRTC1-MAML2 fusion transgene is capable of transforming salivary ductal cells and drives the development of salivary gland tumors in mice.

### Salivary gland tumors arising in the Cre-regulated CRTC1-MAML2 transgenic mouse model exhibit histological characteristics of human MEC.

**Figure 3:**
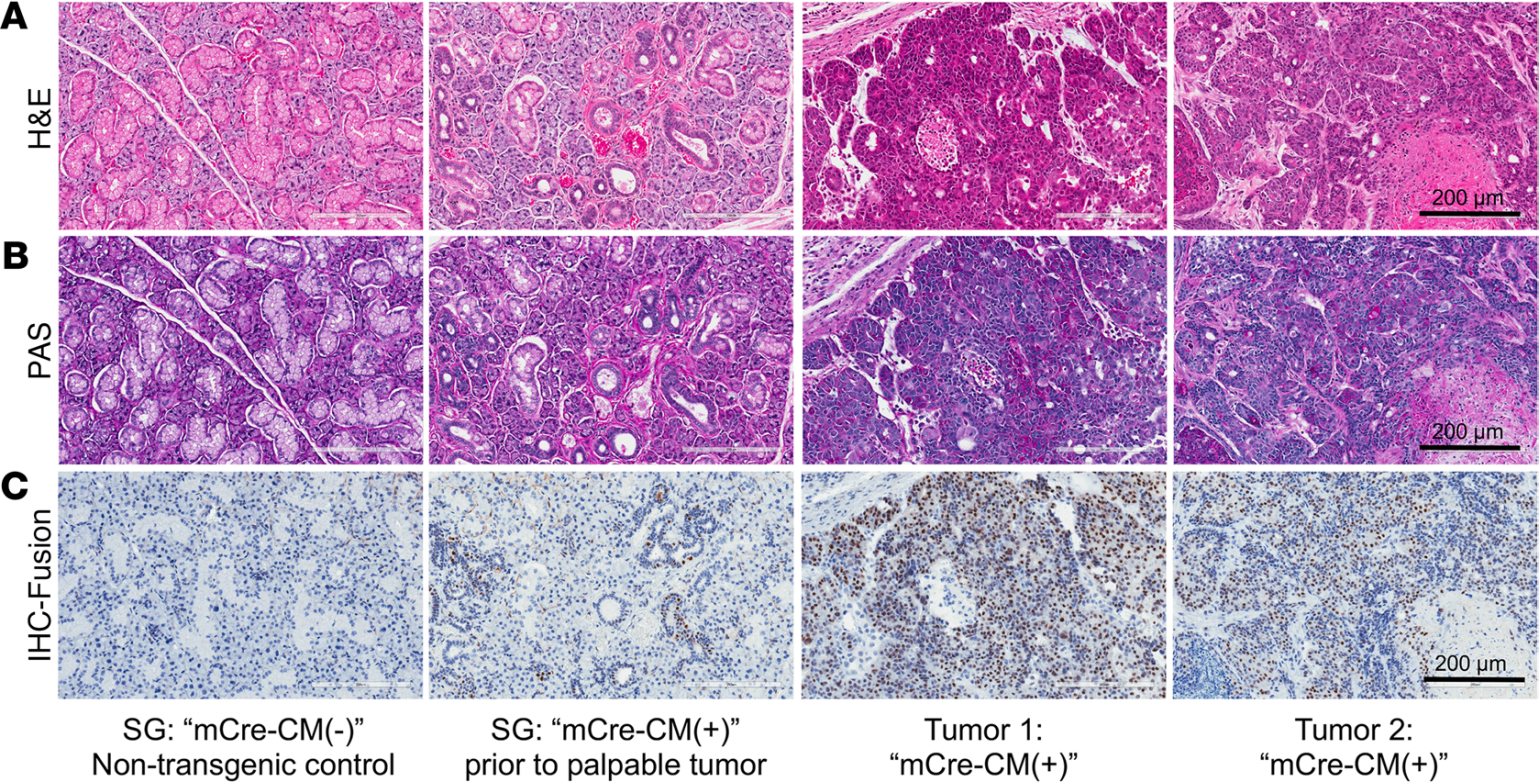
The CRTC1-MAML2 fusion–induced murine salivary gland tumors displayed a characteristic human MEC histological feature and expressed the CRTC1-MAML2 fusion. (**A**–**C**) Submandibular glands (SMG) from nontransgenic mCre-CM(–) littermate controls and from mCre-CM(+) mice at age of 4 weeks and salivary gland tumors developed from mCre-CM(+) mice were subjected to H&E staining for histological analysis (**A**); PAS staining for detecting mucin-expressing cells (**B**); and IHC staining with anti-MAML2 TAD antibodies for detecting the CRTC1-MAML2 fusion expression (**C**). Scale bars: 200 μm.

To characterize the histological subtype of salivary gland tumors developed in Cre-regulated CRTC1-MAML2 transgenic mice, we performed H&E for histological analysis and Periodic acid–Schiff (PAS) staining for detecting mucins. We observed that salivary gland tumors that developed from both the Line 1 and Line 2 contained a mixture of mucin-secreting cells, epidermoid cells, and intermediate cells with various levels of cyst growth (Figure 3, A and B, and Supplemental Figure 7, A and B), a histological feature characteristic of human MEC. When examining salivary glands prior to the formation of a palpable tumor in mCre-CM(+) mice, we detected abnormal ducts, including hyperproliferative lesions of ductal cells and dysplastic cells (Figure 3 and Supplemental Figure 8). Therefore, the CRTC1-MAML2 fusion–driven tumor development likely results from a multistep process from normal, hyperplasia, carcinoma in situ to carcinoma.

To ascertain the CRTC1-MAML2 transgene expression at the cellular levels within the salivary gland tissues and tumors, we performed IHC analysis using an antibody that recognized the CRTC1-MAML2 TAD domain. Positive staining for the CRTC1-MAML2 transgene expression was detected in some but not all ductal cells of salivary glands in mCre-CM(+) mice prior to the formation of palpable tumors, while it was observed in the majority of cells within the tumors (Figure 3C and Supplemental Figure 7C). Moreover, the CRTC1-MAML2 fusion–positive ductal cells were localized in the regions of hyperplasia and carcinoma in situ (compared with the H&E-stained patterns in Figure 3A). These data demonstrate that the transgenic CRTC1-MAML2 fusion expression in salivary gland ductal cells drives the formation of MEC-like tumors and also suggest that additional sequential somatic events likely cooperate with the CRTC1-MAML2 fusion to promote a multistep tumorigenic process.

### The CRTC1-MAML2–induced murine salivary gland tumors display molecular features of human MEC.

**Figure 4:**
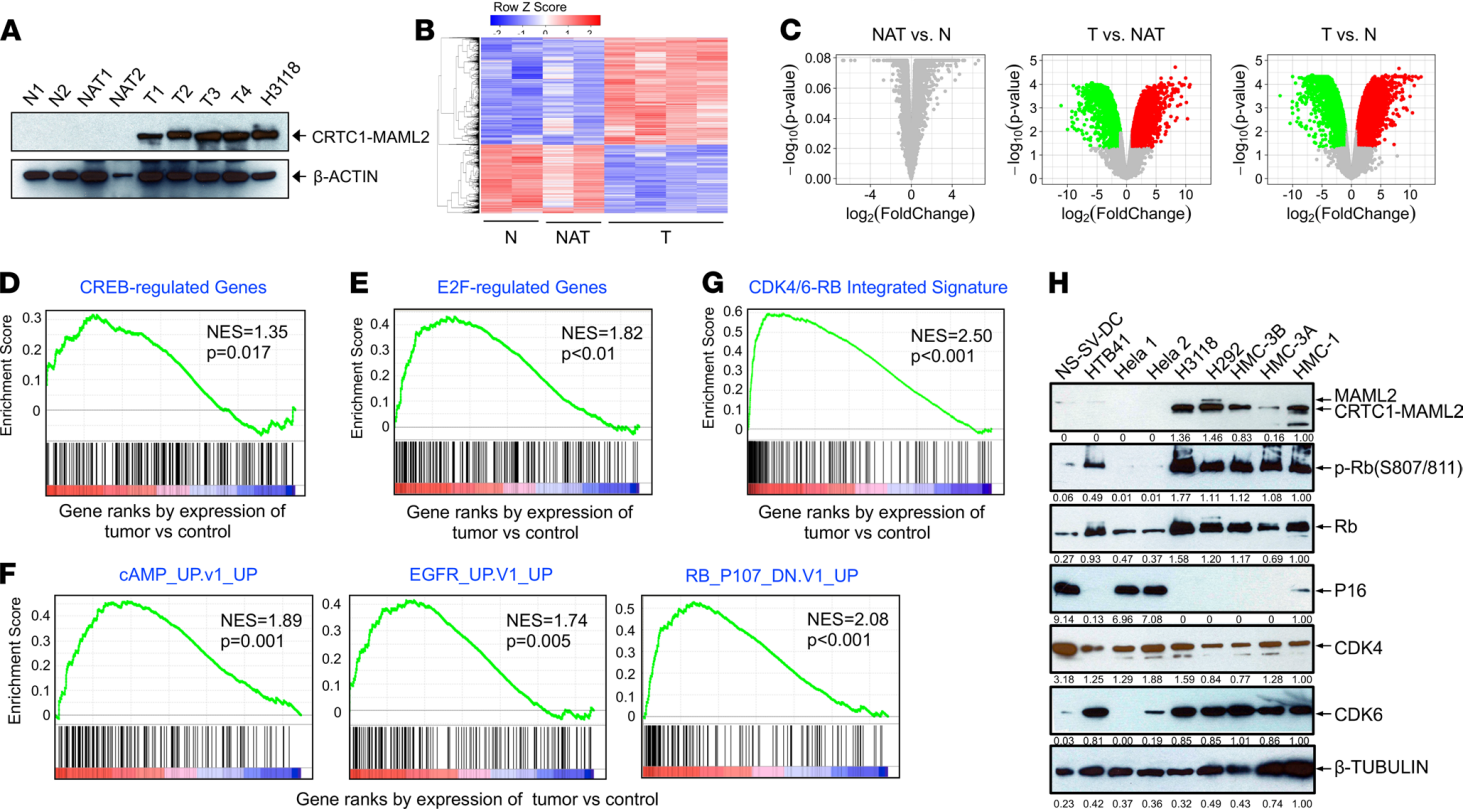
Deregulated cell cycle control in CRTC1-MAML2–induced murine MEC tumors and human CRTC1-MAML2 fusion–positive MEC-derived cell lines. (**A**) Western blotting showed expression of the CRTC1-MAML2 fusion transgene in MEC tumors (T) developed from mCre-CM(+) mice, with barely detectable level in their matched tumor-adjacent salivary glands (NAT) or normal salivary glands (N) from nontransgenic mCre-CM(–) mice. β-Actin was used as a loading control. (**B**) Heatmap shows differentially expressed genes (DEGs) among N, NAT, and T groups. The cutoff criteria were fold-change of ≥ 2 and FDR *P* < 0.05. (**C**) Volcano plots shows differentially expressed genes in NAT versus N, T versus NAT, and T versus N groups. Green and red dots represent downregulated and upregulated genes, respectively. (**D** and **E**) CREB-regulated genes (**D**) and E2F-regulated genes (**E**) were highly enriched in CRTC1-MAML2–induced MEC tumors. (**F**) Representative oncogenic signatures (cAMP-induced, EGFR-induced, and RB1/RBL1 loss–induced) were enriched in the CRTC1-MAML2–induced MEC tumors. (**G**) CDK4/6-RB integrated signature was enriched in fusion-induced MEC tumors. (**H**) Western blot analysis of CRTC1-MAML2, p-Rb, Rb, P16, CDK4, and CDK6 in human fusion–negative cell lines — including a normal human immortalized salivary gland ductal cell line (NS-SV-DC), a submaxillary gland undifferentiated epidermoid carcinoma cell line (HTB-41), a cervical carcinoma cell line from 2 sources (Hela 1 and Hela 2) — and fusion-positive cell lines, including a lung mucoepidermoid cell line (H292), a parotid mucoepidermoid cell line (H3118), MEC cell lines from a palate-derived local recurrent MEC tumors and its lymph node metastasis (HMC-3A and HMC-3B), and a minor salivary gland buccal mucosa-derived mucoepidermoid (HMC-1) cell line. β-Tubulin was used as a loading control.

We next determined whether the CRTC1-MAML2 fusion–induced MEC-like tumors exhibited the molecular features of human CRTC1-MAML2–positive MEC. We harvested the CRTC1-MAML2 fusion–induced salivary gland tumors (T1, T2) and their matched tumor-adjacent normal tissues (NAT1, NAT2), along with normal salivary gland tissues from the control nontransgene carrier littermates (N1, N2). We also included 2 other CRTC1-MAML2–induced salivary gland tumors from separate mice (T3, T4). Western blotting showed that the tumors expressed the CRTC1-MAML2 fusion transgene at a level comparable with that of the endogenous CRTC1-MAML2 fusion in human MEC H3118 cells, whereas the normal and the matched normal adjacent tissues showed undetectable fusion transgene expression (Figure 4A). Known fusion target genes, *Areg* and *Nr4a2*, were validated to be highly expressed in the tumors (Supplemental Figure 9). Gene expression profiling was then carried out, and differentially expressed genes (DEGs) were determined using cut-off criteria of absolute fold change of ≥ 2.0 and FDR *P* ≤ 0.05. As shown in the heatmap and volcano plots (Figure 4, B and C), there were no significantly altered genes between NAT versus N, but there were many DEGs between T versus NAT, and between T versus N groups. The DEGs in CRTC1-MAML2–induced mouse MECs (T versus N) were then compared with the CRTC1-MAML2 target genes we previously identified through the profiling of shRNA-mediated CRTC1-MAML2 depletion versus control human MEC cells (28). This analysis revealed an extensive overlap of these 2 sets of genes: 87 genes showing upregulation in CRTC1-MAML2–induced mouse MEC and downregulation in CRTC1-MAML2–depleted human MEC cells, which include known targets such as *AREG* and *PTGS2*; and 69 genes displaying a reverse pattern (Supplemental Table 1). These results indicate that the *CRTC1-MAML2* transgene is functional in regulating a distinct set of target genes and that mouse CRTC1-MAML2–induced tumors shared many common molecular targets with human fusion–positive MEC cells. Therefore, our transgenic model developed tumors displaying molecular and histological features of human MEC, thus representing a mouse model of human MEC.

### Transcriptomic analysis of CRTC1-MAML2–induced MEC tumors identifies aberrant p16-CDK4/6-RB pathway activity.

Our above data demonstrate that the CRTC1-MAML2 fusion is a key oncogenic driver and a validated therapeutic target in MEC. Since the CRTC1-MAML2 fusion is a nuclear transcriptional cofactor with no enzymatic activity, its direct targeting is difficult to achieve, and the use of the shRNA or gene editing approaches to silence its expression is unfeasible for therapy in current practice. We previously showed that CRTC1-MAML2 induced an autocrine AREG-EGFR signaling and that blocking this aberrant EGFR signaling reduced MEC growth and survival (32), indicating that the identification of the CRTC1-MAML2–induced pathogenic signaling could provide potential rational therapeutic strategies. Due to the multistep feature of the CRTC1-MAML2–driven tumorigenesis and the tumor kinetics with various latency, as well as focal nature of the tumors, we hypothesize that additional sequential signaling events likely cooperate with the CRTC1-MAML2 fusion in tumor progression. To test this hypothesis, we further analyzed the transcriptomic features of the CRTC1-MAML2–induced MEC-like tumors in our mouse model, based on the DRGs in MEC tumors (MEC-DEGs), detected from the comparison group of CRTC1-MAML2–induced tumors (T) versus normal salivary gland tissues (N). Enrichment analysis revealed that MEC-DEGs were highly associated with the top 15 canonical pathways (Table 1), 8 of which were involved in cell cycle (such as cell cycle mitotic, mitotic M-M/G1 phases, DNA replication, G1-S transition, cell cycle checkpoint, and synthesis of DNA), indicating deregulated cell cycle control in CRTC1-MAML2–induced tumors. Other associated pathways were also discovered, such as the CXCR3 pathway, extracellular matrix organization, degradation of the extracellular matrix, amino acid, sugar and nucleotide metabolism, ORC1 removal from chromatin, and events in ERBB4 signaling. Gene set enrichment analysis using the Molecular Signatures Database (MSigDB) curated motif gene sets (C3:TFT) revealed upstream regulators of MEC-DEGs, including CREB (Figure 4D), which was consistent with CRTC1-MAML2 transactivation of the CREB-mediated gene transcription, and E2F (Figure 4E), which was aligned with cell cycle pathway deregulation (Table 1). Using MSigDB oncogenic signatures, we found that MEC transcriptomes were enriched with the oncogenic signatures (Supplemental Table 2), such as cAMP-induced signature (cAMP-up), oncogenic EGFR-induced signature (EGFR-up), and RB1/RBL1 loss–induced signature (RB.107_DN-up) (Figure 4F). Moreover, a CDK4/6-RB integrated signature, identified from a recent pan-cancer molecular analysis (41), was also found to be overrepresented in fusion-induced MEC (Figure 4G). These data were again in agreement with the CREB activation and enhanced EGFR signaling in human MEC, as we previously reported (32). Also, the enrichment of the RB1/RBL1 loss–induced and CDK4/6-RB integrated signatures suggested abnormal p16-CDK4/6-RB pathway activity, which is consistent with an upregulated E2F gene signature (Figure 4E). We next validated enhanced expression of the top 10 genes (*Mcm6*, *Prc1*, *Rrm2*, *Haus8*, *Pole2*, *Ccne2*, *Lig1*, *Dhfr*, *Mcm7*, and *Pcna*) in MEC tumors, which were shared by both the RB1/RBL1 loss–induced and CDK4/6-RB integrated signatures by qPCR (Supplemental Table 3 and Supplemental Figure 10). Furthermore, we observed many phosphorylated RB-stained positive cells in mouse MEC tumors and barely detectable signals in the salivary glands of control nontransgenic littermates (Supplemental Figure 11A), again supporting RB inactivation. The p16-positive cells were detected in both mouse MEC tumors and normal salivary glands, with an increased number of p16-positive cells in the tumors (Supplemental Figure 11B), while the CDK4-positive cells were only detected in mouse MEC tumors and not in normal salivary glands (Supplemental Figure 11C). All these data indicate deregulated cell cycle control in murine CRTC1-MAML2–driven MEC tumors.

A prior study showed unfavorable prognosis for human fusion–positive MEC cases with *CDKN2A* (*p16*) deletions (42). Since p16 is a critical regulator of CDK4/6-RB signaling preventing G1/S cell cycle progression (43, 44), p16 deletion/functional loss likely cooperates with the CRTC1-MAML2 fusion in tumor progression. To determine possible p16-CDK4/6-RB alteration in human MEC, we performed Western blot analysis for p16, RB, CDK4, and CDK6, and we observed that 4 of 5 human fusion–positive MEC cells (H3118, H292, HMC-3B, HMC-3A) has p16 loss and enhanced Rb phosphorylation (Figure 4H). Furthermore, reduced p-RB levels were observed in human MEC cells with shRNA-mediated CRCT1-MAML2/MAML2 depletion, but not with CRISPR/Cas9-mediated MAML2 KO (Supplemental Figure 12); this indicates that the CRTC1-MAML2 fusion impacts the cell cycle, which was consistent with the fusion activity in promoting cell proliferation (28, 32). Since p16 loss is an independent genetic alteration concurrent with the CRTC1-MAML2 fusion in human MEC (Figure 4H), it suggests that the deregulation of p16-CDK4/6-RB is needed to further abrogate the cell cycle control and confer tumor aggressiveness. Therefore, aberrant p16-CDK4/6-RB signaling likely cooperates with the CRTC1-MAML2 fusion in MEC progression, which represents a potential important pathway for therapeutic intervention.

### Coinhibition of aberrant p16-CDK4/6-RB signaling and CRTC1-MAML2–induced autocrine EGFR signaling show synergistic anti-MEC activity in vitro.

**Figure 5:**
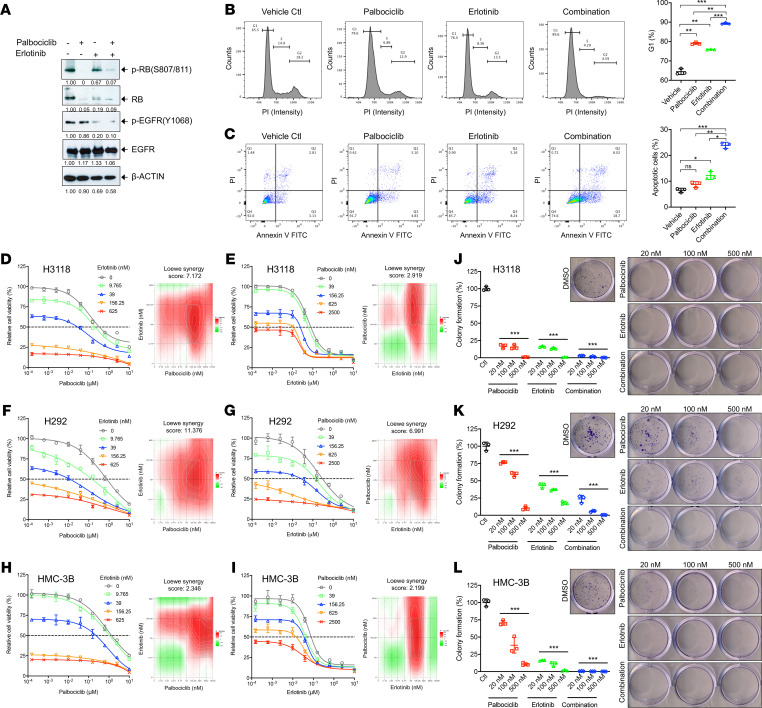
Combination of CDK4/6 inhibitor and EGFR inhibitor led to enhanced inhibition of human MEC cell growth and clonal expansion in vitro. (**A**) Human MEC H3118 cells were treated with Palbociclib (1 μM) and Erlotinib (1 μM) individually or in combination for 24 hours. Cell lysates were harvested for Western blotting analysis. (**B** and **C**) H3118 cells were treated with Palbociclib (200 nM) and Erlotinib (200 nM), individually or in combination. Cell cycle analysis was performed 48 hours after treatment, and cells in G1 were quantified (**B**). Annexin V/PI staining was performed at 72 hours after treatment, and apoptotic cells were quantified (**C**). (**D**–**I**) Human MEC cells, H3118, H292, and HMC-3B, were treated with 9-point dose concentrations of Palbociclib (1:4 dilutions starting from 10 μM) and 4 doses of static Erlotinib concentrations plus vehicle control (0, 9.8 nM, 39 nM, 156 nM, 625 nM) (**D**, **F**, and **H**) or 9-point dose concentrations of Erlotinib (1:4 dilutions starting from 10 μM) and 4 doses of static Palbociclib concentrations plus control (0, 39 nM, 156 nM, 625 nM, 2.5 μM) (**E**,**G**, and **I**) for 72 hours. Synergistic analysis was conducted using SynergyFinder2.0 with the Loewe’s reference model. Loewe’s synergy scores and color scale bars indicate strength of interaction with synergistic effect shown in red in heatmaps. (**J**–**L**) Human MEC cells were seeded at 500 cells per well in 12-well plates and treated with vehicle control, Palbociclib, Erlotinib, or a combination of Palbociclib and Erlotinib the next day (*n* = 3 for each group). After 2-week culture, the colonies were stained with crystal violet and counted by using ImageJ software. The percentage of colony formation was represented as the ratio of the colony number in drug-treated groups to DMSO vehicle control. Data are mean ± SD. One-way ANOVA for multiple comparisons was used to calculate the *P* values (*P < 0.05, **P < 0.01, ***P < 0.001).

In light of our above data suggesting that aberrant p16-CDK4/6-RB signaling serves as a cooperating event for CRTC1-MAML2–driven tumorigenesis, we then tested whether cotargeting of the CRTC1-MAML2 fusion activity and aberrant p16-CDK4/6-RB signaling is effective in blocking MEC. We used Palbociclib and Erlotinib individually or in combination to evaluate their anti-MEC efficacy. Palbociclib is a CDK4/6 inhibitor that blocks heightened cyclin D-CDK4/6 dependence due to deregulated p16-CDK4/6 signaling and hinders hyperphosphorylation of Rb and the subsequent release of E2Fs, which is essential to bypass the G1/S checkpoint (43, 45, 46). Erlotinib is an EGFR tyrosine kinase inhibitor that is expected to interfere with the CRTC1-MAML2–induced AREG-EGFR signaling (32, 47, 48), a major signaling pathway that mediates the CRTC1-MAML2 fusion functions. Western blotting was first performed on protein lysates from human MEC H3118 cells, originally derived from a patient with metastatic MEC of the parotid gland (24), treated with Palbociclib and/or Erlotinib for 24 hours. The result showed that Palbociclib reduced RB phosphorylation and protein stability, that Erlotinib inhibited EGFR phosphorylation, and that the combined treatment reduced both the EGFR and RB phosphorylation (Figure 5A), indicating that these inhibitors caused the respective pathway inhibition. Cell cycle analysis showed that individual treatment of Palbociclib or Erlotinib for 48 hours increased the proportions of cells in the G1 phase, while the combined treatment caused a greater cell cycle arrest (Figure 5B). Annexin V and PI staining analysis at 72 hours after treatment showed that Erlotinib, but not Palbociclib, increased apoptosis, and the combined treatment enhanced the apoptotic effect (Figure 5C). These data indicate that the 2-drug combination could achieve better anti-MEC response. To further determine the drug responses, we next treated 3 fusion-positive MEC cell lines, including H3118 (metastatic parotid MEC), H292 (metastatic pulmonary MEC), and UM-HMC-3B (lymph node metastasis MEC) (24, 49) with multi-dose titrations of Erlotinib and Palbociclib, individually and in combination. Specifically, a range of drug concentrations were set up, including (a) a 9-point dose range of Palbociclib, which was combined with 4 doses of static Erlotinib concentrations plus vehicle control, and (b) a 9-point dose range of Erlotinib, which was combined with 4 doses of static Palbociclib concentrations plus control. CellTiter-Glo luminescent cell viability assays (measuring cellular ATP level as an indicator of cell growth) were performed at 72 hours after treatment, and relative cell viability was presented (Figure 5, D–I). To determine potential synergistic interaction between Palbociclib and Erlotinib, we used SynergyFinder 2.0 (50), which evaluates and visualizes pairwise drug combinations in an interactive analysis. A Loewe’s reference model based on the Isobologram method was used to evaluate the performance of the combination treatment. A Loewe’s synergy score of more than 1, illustrated in red in the heatmap, indicates a synergistic effect of 2-drug combination. As shown in Figure 5, D–I, Erlotinib and Palbociclib combination treatment had synergism in inhibiting MEC cell growth. Moreover, colony formation assays showed that the combination of Palbociclib and Erlotinib resulted in enhanced suppression on MEC cell colony forming activity, as compared with the individual treatments (Figure 5, J–L). Calcusyn combination indices for drug responses in colony assays were shown (Supplemental Figure 13), further indicating synergism of this 2-drug combination in blocking MEC. Therefore, these in vitro results suggest therapeutic efficacy of the combination of Palbociclib and Erlotinib in blocking MEC.

### The combination of CDK4/6 and EGFR inhibitors effectively inhibits the growth of human CRTC1-MAML2 fusion–positive MEC xenografts and mouse CRTC1-MAML2–induced MEC allografts in vivo.

**Figure 6:**
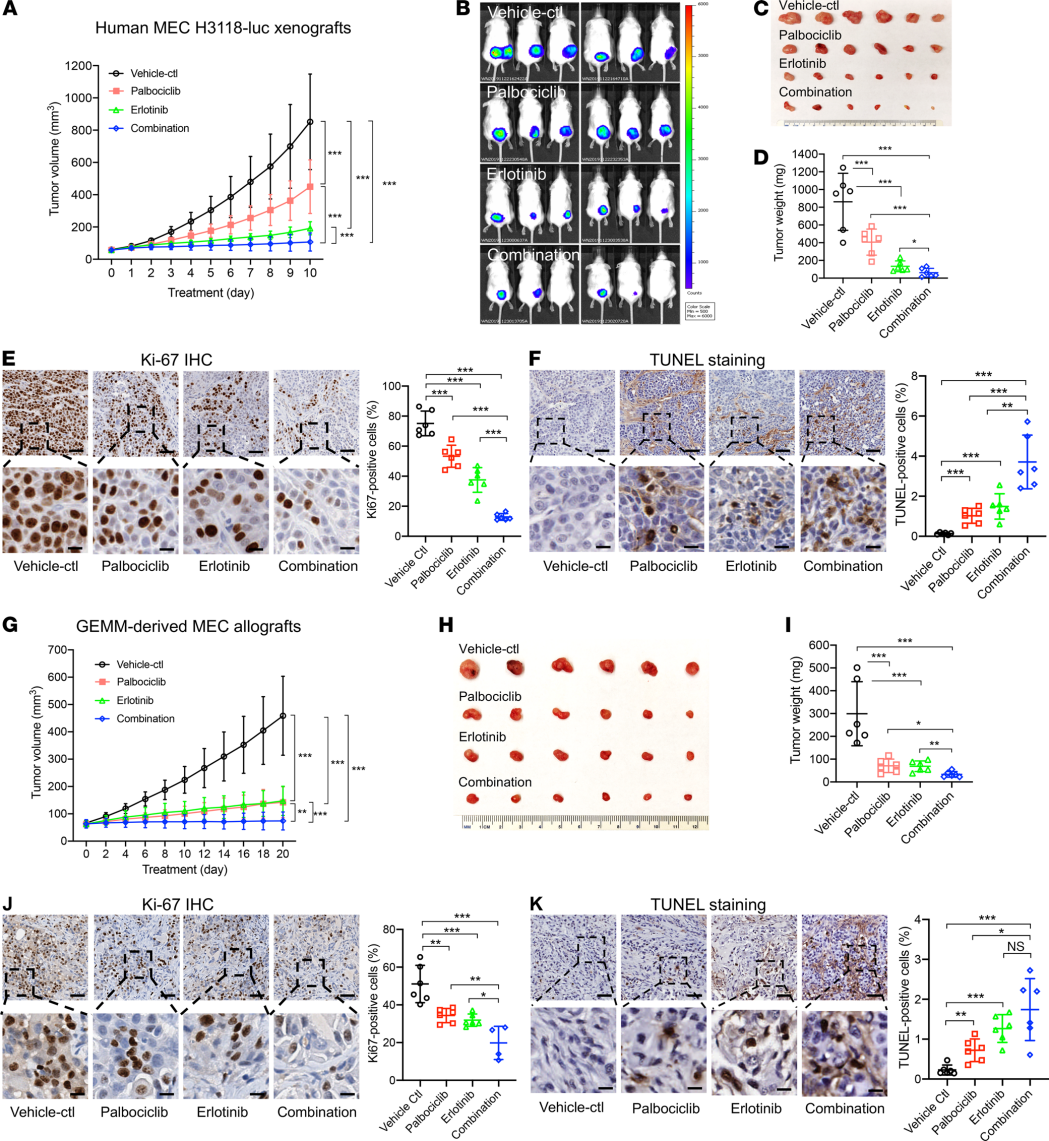
Concurrent CDK4/6 and EGFR inhibition showed enhanced antitumor effect in both human MEC xenograft and mouse CRTC1-MAML2–induced MEC allograft models. (**A**–**F**) Human CRTC1-MAML2 fusion–positive luciferase–expressing MEC (H3118-luc) xenografts were treated with vehicle control (*n* = 6), 25 mg/kg Palbociclib (*n* = 6), 25 mg/kg Erlotinib (*n* = 6), or 25 mg/kg Palbociclib plus 25 mg/kg Erlotinib (*n* = 6) via oral gavage daily for 10 days. The tumors were measured daily after treatment, and the tumor growth curve was represented by tumor volumes. (**B**–**F**) Bioluminescent imaging of the xenograft tumors (**B**), tumor images (**C**), and tumor weights (**D**) were presented on the final day of treatment. (**E** and **F**) Representative images of IHC staining of Ki-67 (**E**) and TUNEL (**F**) on xenograft tumor sections for vehicle control, Palbociclib, Erlotinib, or their combination. (**G**–**K**) The CRTC1-MAML2–positive mouse MEC allografts were treated with vehicle control (*n* = 6), 25 mg/kg Palbociclib (*n* = 6), 25 mg/kg Erlotinib (*n* = 6), or the 2 inhibitors in combination (1:1 ratio) (*n* = 6) via oral gavage daily for 20 days. The tumor volumes were measured every other day after treatment (**G**), tumor images (**H**), and weights (**I**) at the endpoint were presented. Representative images of Ki-67 IHC (**J**) and TUNEL staining (**K**) of allograft tumor sections were shown. The staining-positive cells in the tumor sections were quantified by ImageJ. Data are mean ± SD. One-way ANOVA test was used for multiple comparisons (*P < 0.05, **P < 0.01, ***P < 0.001). Scale bars: 100 μm (upper panels), 25 μm (lower panels).

We next evaluated the antitumor efficacy of Palbociclib and Erlotinib, individually and in combination, using human MEC xenograft and Genetically engineered mouse model–derived (GEMM-derived) MEC allograft models. First, NOD.SCID mice were s.c. injected with human MEC H3118 cells, randomly divided into 4 groups when tumors reached approximately 50 mm^3^, and then orally administered with the following agents: vehicle control (*n* = 6); 25 mg/kg Palbociclib (*n* = 6); 25 mg/kg Erlotinib (*n* = 6); and 25 mg/kg Palbociclib plus 25 mg/kg Erlotinib (*n* = 6). The drug doses for these 2 inhibitors were selected based on the published studies (51, 52). We observed that Erlotinib, Palbociclib, or their combination caused a significant inhibition on tumor growth, as indicated by reduced tumor growth, size, and weight, as compared with the vehicle control (Figure 6, A–D). No significant changes in body weight were observed. Moreover, the combination group showed a better antitumor effect, as compared with either monotherapy cohorts. The IHC analysis of xenograft tumors for the Ki-67–positive proliferating cells and the TUNEL assay for the apoptotic cells showed similar trends (Figure 6, E and F).

We also tested drug responses of GEMM-derived allografts in immunocompatible mice, since our GEMM mouse model mCre-CM(+) also developed skin cyst phenotypes and had variable tumor onset (Figure 2E and Supplemental Figure 5). We engrafted GEMM-derived mouse MEC tumor cells s.c. in immunocompetent, transgene-negative control mCre-CM(–) mice and performed drug treatment as described above. Again, the combination of CDK4/6 inhibitor Palbociclib and EGFR inhibitor Erlotinib showed a better antitumor efficacy (Figure 6, G–K). Also, no significant changes in body weight for the drug-treated and control cohorts were observed. Therefore, the data from both human MEC xenografts and GEMM-derived MEC allografts indicate that the combined inhibition of the CRTC1-MAML2 fusion–induced EGFR signaling and the secondary, altered pathway p16-CDK4/6-Rb signaling effectively blocks MEC in these preclinical MEC models.

## Discussion

There are no effective systemic treatments for unresectable, recurrent, or metastatic MEC. Clinical progress has been hindered by a limited understanding of MEC pathogenesis, lack of preclinical models, and its status as a rare disease with limited opportunities for investigational clinical trials. Although previous studies show that the *CRTC1-MAML2* fusion oncogene is a recurrent somatic mutational event in MEC, direct evidence for its role as an oncogenic driver for MEC tumorigenesis and maintenance in vivo remained lacking. In the present study, we engineered new models, including the first GEMM mouse model of MEC to our knowledge and human MEC cells with Dox-inducible CRTC1-MAML2 shRNA. These models have enabled us to define the role of the CRTC1-MAML2 fusion in MEC establishment and maintenance and prove that the CRTC1-MAML2 fusion is the major oncogenic driver for MEC initiation and maintenance. We also identified altered p16-CDK4/6-RB activity as a likely cooperative event for CRTC1-MAML2–induced tumorigenesis in vivo and showed that a combination of EGFR and CDK4/6 inhibition effectively blocked MEC, thus revealing a potential effective treatment for patients with MEC.

Although our prior studies suggest a critical role of the CRTC1-MAML2 fusion expression in the growth and survival of human MEC cells in vitro and in xenograft studies (31, 32), its role in maintaining established MEC tumors in vivo has not been validated. In this study, we generated a human MEC cell model that displayed Dox-induced robust and regulatable shRNA-mediated silencing of the CRTC1-MAML2 fusion and observed that Dox treatment caused potent growth inhibition of established MEC xenograft tumors. These data demonstrate that MEC is critically dependent on the CRTC1-MAML2 fusion expression for sustained growth; therefore, the CRTC1-MAML2 fusion represents a validated target for therapeutic intervention. Further critical molecular details of CRTC1-MAML2 fusion functions are essential for the development of new treatments. Our engineered cell model allows tightly controlled CRTC1-MAML2 shRNA expression and, thus, will be useful for modeling pathways and networks, as well as probing the mechanisms of action and tumor phenotypes during MEC progression.

In addition to tumor development in major and minor salivary glands, the CRTC1-MAML2 fusion is also associated with tumors with MEC-like histological patterns arising throughout the body, suggesting that it is a unifying early event in MEC pathogenesis. CRTC1-MAML2 (and, to lesser extent, CRTC3-MAML2) has been detected in the majority of human MEC cases, and MAML2 break-apart FISH currently serves as a biomarker for MEC diagnosis. In contrast to the detection of MAML2 rearrangement, there are very limited additional genetic changes from recent whole-exome sequencing, copy number, and translocation analyses (53–55). Our prior work provided in vitro evidence that the CRTC1-MAML2 fusion is capable of transforming epithelial cells (24, 26). However, whether this fusion drives the formation of MEC in vivo had not been proven. Here, we developed a Cre-regulated CRTC1-MAML2 transgenic mouse model and showed that Cre-induced expression of the transgenic CRTC1-MAML2 fusion in salivary glands, either by crossing with MMTV-Cre mice or direct AAV-Cre transduction, resulted in the development of salivary gland tumors that closely mimicked the histological and molecular features of human MEC. We typically observed that single palpable tumors arose among the transgenic carrier animals over the 3 to 9-month observation period in our mCre-CM(+) model. The *CRTC1-MAML2* transgene was found expressed in some but not all ductal cells within the salivary glands, which was consistent with the mosaic Cre expression in MMTV-Cre line as previously reported (56). This mosaic expression pattern of the CRTC1-MAML2 transgene in salivary glands better mimics the situation in which somatic oncogenic changes usually occur in a few somatic cells during human cancer development. Importantly, the CRTC1-MAML2 fusion–expressing cells were colocalized in the hyperproliferative, abnormal ductal regions at early stage and within the tumors, strongly supporting that the CRTC1-MAML2 fusion–expressing cells give rise to tumors through a multistep process from normal, precancerous, to overt tumors. These data indicate that the CRTC1-MAML2 fusion is an initiating event in MEC pathogenesis.

The focal nature of tumors and tumor latency suggests that the CRTC1-MAML2 fusion is required but that secondary cooperating abnormalities are also needed for facilitating MEC development and progression. Importantly, human fusion–positive MEC cases show a broad spectrum of differentiation and biological aggressiveness from low-grade to intermediate- or high-grade tumors having a very low survival rate (7–9). The CRTC1-MAML2 fusion likely regulates a common transformation event and cooperates with additional events leading to tumor progression or higher grade with propensity for metastases. To study the CRTC1-MAML2–induced downstream signaling, as well as its cooperative signaling events in MEC tumors, we performed expression profiling analysis of mouse MEC–like tumors. We observed upregulated CREB-mediated transcription associated with DEGs in mouse MEC tumors (MEC-DEGs), and this was consistent with the function of the CRTC1-MAML2 fusion in constitutive activation of the CREB transcriptional program. Notably, MEC-DEGs were found to be highly enriched with genes associated with cell cycle and G1-S checkpoint, suggesting that abrogation of G1-S checkpoint is a crucial cooperating event for the CRTC1-MAML2 fusion–induced tumorigenesis. These data were in line with the efficient transformation of E1A-immortalized RK3E epithelial cells (24, 26) and the lack of p16 expression in the majority of established human MEC cell lines we tested (Figure 4H). Moreover, a prior study revealed unfavorable prognosis for human fusion–positive MEC cases with CDKN2A (p16) deletions (42). Since deregulated p16-CDK4/6-RB signaling cooccurs with the CRTC1-MAML2 fusion, we propose a model in which deregulated p16-CDK4/6-RB signaling serves as a cooperating event in the progression of CRTC1-MAML2–driven tumors. Specifically, the CRTC1-MAML2 fusion is an initiating oncogenic event for establishing MEC by transforming permissive cells within salivary glands and endowing cells with the proliferative and survival advantages. Subsequent alterations, such as the loss of p16, causes CDK4/6-RB activity and maximizes cell growth. The malignant phenotypes of MEC consequently become dependent on both the fusion and deregulated CDK4/6-RB activities.

Based on this model, we reasoned that targeting 2 key signaling events in MEC development and progression, namely the CRTC1-MAML2 fusion activity and deregulated p16-CDK4/6-RB signaling, might be effective in blocking MEC. Previously, we identified that the CRTC1-MAML2 fusion transactivates expression of the EGFR ligand AREG and induced an autocrine AREG-EGFR signaling loop that fuels MEC growth and survival (32). The inhibition of EGFR signaling via anti-EGFR antibodies (Cetuximab) blocked MEC growth (32). Therefore, we investigated cotargeting of aberrant p16-CDK4/6-RB activity and CRTC1-MAML2–induced EGFR signaling, by respective FDA-approved drugs CDK4/6 inhibitor Palbociclib and EGFR inhibitor Erlotinib. Our data show that this combination was effective in blocking MEC growth in human MEC xenografts, as well as GEMM-derived MEC allografts. It should be noted that drug treatment of human MEC xenograft tumors was carried out only for 10 days due to aggressive tumor growth for the control group. Studying tumor growth of drug-treated mice over a longer period and different doses of drugs is needed to identify optimal tumor inhibition. Nevertheless, our data strongly suggest the combination of CDK4/6 inhibitor and EGFR inhibitor represents a promising therapeutic strategy for patients with MEC. In the future, comparisons of fusion-expressing cells at various stages of MEC tumorigenesis at single-cell resolution will enable systemic identification of cooperating events for CRTC1-MAML2 fusion–mediated MEC development and progression; such efforts will provide comprehensive mechanistic insights and uncover new effective treatment approaches.

In summary, we established a GEMM model of MEC and a MEC cell model with inducible CRTC1-MAML2 knockdown, and we provided direct genetic proof for the CRTC1-MAML2 fusion as a key driver of MEC initiation and maintenance. We also identified altered p16-CDK4/6-RB signaling as a cooperating event for CRTC1-MAML2–induced tumorigenesis and showed that FDA-approved drugs, the CDK4/6 inhibitor Palbociclib that targets aberrant p16-CDK4/6 activity and the EGFR inhibitor Erlotinib that targets CRTC1-MAML2–induced EGFR signaling, were effective in blocking MEC growth. This Cre-regulated CRTC1-MAML2 transgenic mouse model is the first GEMM model to our knowledge that is highly relevant to human MEC. Our study, thus, offers valuable preclinical models for the further probing of CRTC1-MAML2 transformation and the interplay of tumor-host interactions, as well as the identification of new strategies for clinical management of MEC. These data may also prove useful for studying strategies to block aberrant CRTC signaling in other common adult malignancies, such as LKB1-deficient lung cancers and melanomas.

## Methods

### Cell culture.

Human CRTC1-MAML2–positive MEC cell lines (24, 49), including H3118 (metastatic parotid MEC), H292 (metastatic pulmonary MEC), UM-HMC-3A (palate-derived local recurrent MEC), and HMC-3B (lymph node metastatic MEC) from the same patient, and UM-HMC-1 (a minor salivary gland buccal mucosa-derived MEC) were previously described (24, 49). Fusion-negative cancer cells, including HTB-41 (submaxillary gland undifferentiated epidermoid carcinoma cell line) and Hela (cervical carcinoma cell line) were originally obtained from ATCC and 293FT from Thermo Fisher Scientific. All of these cell lines were maintained in DMEM (Mediatech) supplemented with 10% inactivated FBS (Lonza) and 1% penicillin/streptomycin (Mediatech). UM-HMC-3A, UM-HMC-3B, and UM-HMC-1 were provided by Jacques Nör (University of Michigan, Ann Arbor, Michigan, USA). NS-SV-DC (normal human immortalized salivary gland cell line) (57) was cultured with the Keratinocyte-SFM medium with L-Glutamine, EGF, and BPE (Thermo Fisher Scientific). Dox-inducible shRNA cell lines that we generated in this study were cultured in DMEM supplemented with 10% inactivated tetracycline-free FBS (Atlanta Biologicals). Cells were maintained in the humidified incubator at 37°C with 5% CO_2_.

### Establishment of human MEC cells expressing Dox-inducible CRTC1-MAML2 shRNAs.

The Dox-inducible lentiviral expression vector FH1tUTG was provided by Marco J. Herold at The Walter and Eliza Hall Institute, Australia (34). Two oligonucleotides containing CRTC1-MAML2–targeting shRNA sequences were annealed and cloned into the BsmbI/XhoI sites of the FH1tUTG vector which constitutively expresses eGFP. The following oligo sequences were used: ishCRTC1-MAML2 oligo 1 (sense, 5′-TCCCCCCTGTCTAAACTCCAGGATACTCGAGTATCCTGGAGTTTAGACAGGGTTTTTC-3′, and antisense, 5′-TCGAGAAAAACCCTGTCTAAACTCCAGGATA CTCGAGTATCCTGGAGTTTAGACAGGG-3′); ishCRTC1-MAML2 oligo 2 (sense, 5′- TCCCCCCAAAGCAATTGTTAGCAAACTCGAGTTTGCTAACAATTGCTTTGGGTTTTTC-3′, and antisense, 5′-TCGAGAAAAACCCAAAGCAATTGTTAGCAAA CTCGAGTTTGCTAACAATTGCTTTGGG-3′). The resulting FH1tUTG_ishCRTC1-MAML2 constructs, together with packaging plasmid psPAX2 and envelope-expressing plasmid pMD2.G, were transfected to HEK293FT cells using Effectene transfection reagent (Qiagen) to make lentiviruses. The virus-containing cell culture supernatants were collected at 48 hours and 72 hours after transfection and used to infect target MEC H3118 cells on 2 consecutive days. The transduced cells were sorted for GFP-expressing cells using a flow cytometer, and they were then subjected to singe-cell cloning by serial dilutions in 96-well plates. The single-cell clones were later expanded and screened for shRNA inducibility with Dox treatment. In brief, H3118-fusion ishRNA cells were seeded at 5 × 10^5^ cells per well in 6-well plates overnight and then cultured with or without Dox (1 μg/mL) for 72 hours. Cells were collected and analyzed by Western blotting and qPCR assays to determine the CRTC1-MAML2 knockdown efficiency.

### Dox treatment in vivo.

H3118-fusion ishRNA cells (1 × 10^6^) were mixed with 100 μL of Matrigel (BD Biosciences , 354230)/PBS (Thermo Fisher Scientific, 10010-023) (1:1) and injected s.c. into the right flank of 8- to 12-week-old NOD/SCID mice (The Jackson Laboratory). Mice were randomly separated into 2 groups and fed with control diet (Bio-Serv, S4207, *n* = 5) or 200 mg/kg Dox chow diet (Bio-Serv, S3888, *n* = 6) immediately after the implantation of tumor cells. In a second experiment, mice were provided with control diet (*n* = 5) or Dox chow when the volumes of xenografts reached approximately 50 mm^3^ (*n* = 6) or approximately 100 mm^3^ (*n* = 6) after implantation. The tumors were measured daily with a Dial caliper and tumor volumes were calculated based on this formula: tumor volume = (length × width^2^) × 0.5.

### Generation of Cre-regulated CRTC1-MAML2 transgenic mice and genotyping.

A approximately 3.6 kb of *Nhe*I*-Not*I human CRTC1-MAML2 cDNA fragment, consisting of the Kozak-ATG-FLAG epitope sequence in frame with the entire open reading frame of CRTC1-MAML2, followed by approximately 400 bp of 3′UTR, was cloned into the pCBR vector (a gift of Sun Tao, Cornell University Weill Medical College, New York, New York, USA) (58) to obtain the transgenic construct pCBR-LSL-CM. This construct was verified by DNA sequencing and confirmed for Cre-dependent transgene expression in mammalian HEK293T cells through cotransfection with a Cre expression vector pCAGIG-Cre (a gift of Sun Tao). Subsequently, the 8.3 kb fusion transgene fragment (LSL-CM) was purified after the digestion of pCBR-LSL-CM plasmid DNA with *Kpn*I to remove plasmid vector backbone, which was used for microinjection into pronuclei of fertilized eggs from FVB/J mice in our Mouse Models Core (University of Florida). The transgenic mice (LSL-CM) were identified by PCR amplification of tail genomic DNA with the CRTC1-MAML2 primers (forward, 5′-TTCGAGGAGGTCATGAAGGA-3′; reverse, 5′-TTGCTGTTGGCAGGAGATAG-3′). Transgenic mice were maintained by crossing with WT FVB/J mice (001800, The Jackson Laboratory).

### Transgene copy number calculation.

PCR assays were performed with 0.1, 1, 10, 100, and 1000 copies of the pCBR-LSL-CM transgene construct, and a standard curve was established. Since the haploid content of a mammalian genome is approximately 3 × 10^9^ bp, it was estimated that 100 ng of the tail genomic DNA was equivalent to 0.277 pg of the purified transgenic LSL-CM fragment (8.3 kb). The transgene copy numbers of the LSL-CM lines were determined by performing qPCR of tail DNAs for the Ct values followed by inferring the gene copy numbers from the established standard curve.

### Crossing Cre-regulated CRTC1-MAML2 transgenic mice with Cre lines.

The Cre-regulated CRTC1-MAML2 transgenic mice were crossed with homozygous transgenic MMTV-Cre mice (Tg[MMTV-cre]4Mam/J, 003553, The Jackson Laboratory) to induce expression of the CRTC1-MAML2 fusion transgene in salivary gland. Mice carrying the fusion transgene were identified by PCR as described above. Nontransgenic littermates were used as negative controls.

The Cre-regulated CRTC1-MAML2 transgenic mice were also crossed with the Dcpp1tm1.1 (cre/ERT2)Ovi (028731, Jackson Laboratory; designated as “dCre-ERT2”), and Piptm1.1(cre/ERT2)Ovi (023201, Jackson Laboratory; designated as “pCre-ERT2”). dCre-ERT2 is a tamoxifen–inducible (TAM-inducible) Cre strain in which a fusion of Cre to a mutant form of human estrogen receptor (ERT2) was inserted right after the initiation ATG codon of *Dcpp1* exon 1, and its TAM-induced Cre expression was specific to sublingual serous demilune cells and intercalated duct cells in parotid glands (38). pCre-ERT2 is a TAM-inducible Cre strain in which exon 1 of *Pip* gene is replaced by *Cre-ERT2* fusion, and its TAM-induced Cre expression was specific to acinar cells in submandibular glands (38). TAM (75 mg/kg body weight) was i.p. injected to mice at about 4 weeks of age once daily for 3 consecutive days.

### Transduction of mouse salivary glands with AAV5-Cre-GFP viruses.

The AAV5-Cre-GFP viruses (7018, Vector BioLabs) that express Cre recombinase and eGFP marker independently driven by their own CMV promoters were delivered into submandibular glands by retrograde ductal administration (40). In brief, mice were anesthetized with ketamine (60 mg/mL, 1 μL/g body weight; Phoenix Scientific) and xylazine (8 mg/mL, 1 μL/g body weight; Phoenix Scientific) through intramuscular injection and then injected intramuscularly with atropine (0.5 mg/kg body weight; MilliporeSigma) to reduce salivary flow. Mice were subsequently infused with 50 μL of AAV5-Cre-GFP viruses (4.5 × 10^8^ viral particles in isotonic saline) through a tapered cannula inserted into an orifice of the main excretory duct.

### RNA isolation and qPCR.

Tissues or tumors were processed for RNA isolation after snap freezing in liquid nitrogen. Total RNA was extracted using Trizol reagent (Invitrogen) and purified by RNAeasy Mini Column (Qiagen) and NucleoSpin RNA Column. RNA was reversely transcribed into cDNA using a GeneAmp RNA PCR kit (Applied Biosystems). qPCR was performed using the StepOne Real-Time PCR System (Applied Biosystems) with the SYBR Green PCR Core Reagents Kit (Applied Biosystems). Gapdh was used as an internal control to normalize gene expression levels. The primer sequences were listed in Supplemental Table 4.

### Western blotting analysis.

Tissues or tumors were processed for protein isolation after snap freezing in liquid nitrogen. Protein extracts were prepared and subjected to immunoblotting as previously described (32). The following antibodies were used: anti-MAML2 TAD (catalog 4618), anti–phospho-RB (Ser807/811, catalog 8516), anti–phospho-EGF receptor (Tyr1068, catalog 3777), and anti-EGFR (catalog 2232) from Cell Signaling Technology; anti-FLAG (catalog PAI-984B) from Thermo Fisher Scientific; anti-RB (catalog 554136) from BD Biosciences; anti–β-actin (catalog 5316) from Sigma-Aldrich; anti-CDK4 (catalog sc-260), anti-CDK6 (catalog sc-177), and anti-P16 ( catalog sc-56330) from Santa Cruz Biotechnology; and anti–β-tubulin (catalog 1799-1) and anti-GAPDH (catalog 2251-1) from Epitomics. The protein bands on Western blots were quantified by using ImageJ software (NIH).

### H&E staining, PAS staining, and IHC.

The excised tissues were fixed in 4% paraformaldehyde (PFA) for 24 to 48 hours, transferred to 70% ethanol, processed, and embedded in paraffin. Sections were cut at 4 μm and mounted on slides for H&E and Periodic acid Schiff (PAS) staining. For IHC, sections were incubated with the primary antibodies for 60 minutes followed by incubation with immPRESS goat anti-rabbit or goat anti-mouse IgG polymer (catalogs MP-7602 and MP-7601, Vector BioLabs) for 30 minutes. The antigen-antibody complexes were detected using the ImmPACT DAB peroxidase (HRP) substrate (Vector BioLabs) and counterstained with CAT hematoxylin counterstain (Biocare) according to the manufacturer’s instructions. The following primary antibodies used for the IHC analysis included MAML2 TAD antibody (1:100, Bethyl IHC-00446), Ki-67 antibody (1:100, Dako, Agilent msxhu, 7240), cleaved caspase-3 antibody (1:400, Cell Signaling Technology, 9664), phospho-RB antibody (1:400, Cell Signaling Technology, 8516), Cdk4 antibody (1:300, Santa Cruz Biotechnology Inc., sc-166373), and P16 antibody *(*1:50, Santa Cruz Biotechnology Inc., sc-1661). TUNEL assays were performed using ApopTag Peroxidase In Situ Apoptosis Detection Kit (EMD Millipore, S7100) according to the manufacturer’s instructions. The stained tissue sections were scanned and digitized using Aperio Imagescope (Leica).

### Microarray gene expression profiling.

RNA samples were subjected to gene expression profiling using the Mouse LncRNA/mRNA Arrays v3.0 (8 × 60K, Arraystar Inc). The microarray data were deposited in NCBI Gene Expression Omnibus (GEO; GSE143264). Genes with an absolute fold change of *≥* 2 and FDR *P* <0.05 were considered as significantly DEGs. Gene set enrichment analyses were performed against the MSigDB (v6.2) as previously described (28).

### Cell viability assays and synergy analysis.

Exponentially growing cells (H3118, H292, and HMC-3B) were seeded in 96-well plates at 2000 cells/well in 100 μL of medium overnight and treated with Palbociclib (PD0332991; MedChemExpress) and Erlotinib (LC Laboratories), individually and in combination. Briefly, a range of drug concentrations were set up, including (a) a 9-point dose range of Palbociclib (1:4 dilutions starting from 10 μM) plus vehicle control, which were combined with 4 doses of static Erlotinib concentrations (9.8 nM, 39 nM, 156 nM, 625 nM) plus vehicle control; and (b) a 9-point dose range of Erlotinib (1:4 dilution starting from 10 μM) plus vehicle control, which were combined with 4 doses of static Palbociclib concentrations (9.8 nM, 39 nM, 156 nM, 625 nM) plus control for 72 hours. Synergy analysis was performed using the web-based application SynergyFinder2.0 (https://synergyfinder.fimm.fi), and the synergy score was determined using the Loewe’s reference model (50).

### Colony formation assays.

MEC cells were seeded at 500 cells per well in 12-well plates overnight and treated with Palbociclib and Erlotinib alone or in combination at the indicated concentrations. A total of 0.1% DMSO was used as a control. Culture medium was changed every 4 days with fresh drugs. At 2 weeks after treatment, the colonies were fixed with 4% PFA for 15 minutes at room temperature and stained with 0.5% crystal violet for 1 hour. The colony number was counted using ImageJ (NIH; version 1.51J8) (59). The percentage of colony formation was presented as the ratio of the colony number in drug-treated groups, compared with DMSO-treated controls. The combination index values for combined treatment were analyzed by Calcusyn software (Biosoft) using Chou-Talalay method (60).

### Tumor responses to drug treatment in vivo.

Drug treatments were conducted in 2 sets of experiments using xenograft and allograft models. For the human MEC xenograft model, immunocompromised NOD/SCID mice (NOD.CB17-Prkdc^scid^/J, The Jackson Laboratory), aged 8–12 weeks, were s.c. injected with human luciferase-expressing MEC H3118 cells (1 × 10^6^ cells in 100 μL Matrigel [BD Biosciences]/PBS [v/v, 1:1]). For the allograft model, immunocompatible mCre-CM(–) mice, which carried only MMTV-Cre but no fusion transgene, were s.c. injected with cell suspensions from primary mouse MEC tumors developed from mCre-CM(+) mice. Specifically, mouse MEC tumors were resected, cut into small pieces, and digested with collagenase II (1.15 mg/mL) and hyaluronidase (2 mg/mL) for 90 minutes at 37°C. The tumor cell suspensions were sequentially filtered through 100 μM and 40 μM filters (BD Biosciences), and 1 × 10^6^ tumor cells were suspended in 100 μL Matrigel/PBS (v/v, 1:1) was s.c. injected per mouse.

Mice bearing MEC xenografts or allografts of approximately 50 mm^3^ were randomly divided into 4 cohorts and treated with vehicle control (50 mM sodium lactate, pH = 4, *n* = 6), Palbociclib (25 mg/kg in 50 mM sodium lactate, *n* = 6), Erlotinib (25 mg/kg in 10% Captisol, *n* = 6), or these 2 inhibitors in combination (ratio=1:1, *n* = 6) via oral gavage daily. Tumors were measured every day using a Dial caliper. Tumor volumes were calculated by the use of the formula: (length × width^2^) × 0.5. Body weights were measured daily. At the end point, bioluminescence imaging was performed (for xenografts) as previously described (32), tumors were resected, and tumor weights were measured.

### Statistics.

Data were analyzed for statistical difference using unpaired, 2-tailed unpaired Student’s *t* test for 2-group comparisons or 1-way ANOVA for multiple comparisons (GraphPad Prism 8.0). Statistical significance was defined as a *P* value of less than 0.05. Graphs show mean ± SD.

### Study approval.

Mouse studies were approved by the IACUC of the University of Florida and conducted in accordance with the NIH guidelines for the care and use of laboratory animals.

## Author contributions

ZC and WN performed in vitro and in vivo experiments and wrote the manuscript. MDH assisted with AAV injection to salivary glands in mice. YS performed IHC analysis. JLL and JWL performed data analysis. SL generated the transgenic construct. XZ performed in vitro experiments and assisted with maintaining mouse colonies and with genotyping. MEL, SZ, CL, JL, JDG, and FJK provided reagents, suggestions, data interpretation, and manuscript editing. LW designed and directed the study and wrote the manuscript.

## Supplementary Material

Supplemental data

## Figures and Tables

**Table 1:**
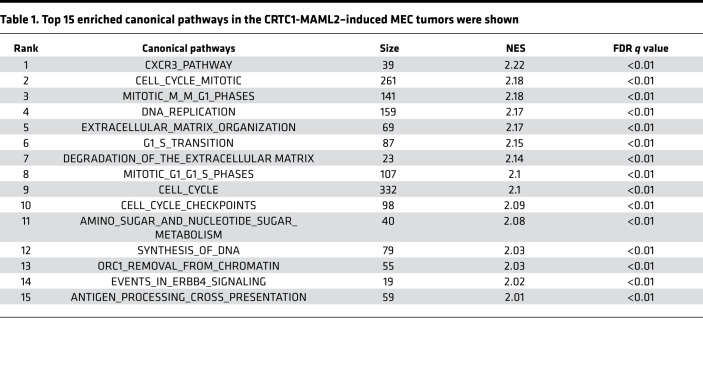
Top 15 enriched canonical pathways in the CRTC1-MAML2–induced MEC tumors were shown

## Version 1 04/08/2021

Electronic publication
