# Supplementary material for: The CRTC1-MAML2 fusion is the major oncogenic driver in mucoepidermoid carcinoma
Source: JCI Insight. 2021 Apr 8;6(7):e139497. doi: 10.1172/jci.insight.139497 (PMC8119194; doi:10.1172/jci.insight.139497)
Supplement: Supplemental data [file jciinsight-6-139497-s024.pdf]

Supplemental Information

Outline……………………………………………………………………………………………………..…1

Supplemental Figures (S1-S13)

Figure S1: A mouse strain carrying a CRTC1-MAML2 knock-in allele under the *Crtc1* natural regulatory control (mimicking the t(11;19) genetic abnormality in human MEC) died after birth................................ 2

Figure S2: The transgene construct, genotyping, and transgene copy numbers in three Cre-regulated CRTC1-MAML2 transgenic mouse lines were shown. 3

Figure S3: Western blotting analysis of the CRTC1-MAML2 fusion transgene expression in salivary gland tumors developed from Line 1 and Line 2 mCre-CM (+) mice…………………………………………..….4

Figure S4: Western blot analysis of the CRTC1-MAML2 transgene expression in various tissues of the mCre;CM(+) mouse line.. 5

Figure S5: The mCre-CM1(+) mice developed ear cysts with CRTC1-MAML2 fusion expression ………...6

Figure S6: The Cre-regulated CRTC1-MAML2 transgenic line, after being crossed with Dcpp1-Cre-ERT2 or Pip-Cre-ERT2 transgenic mice, expressed low levels of the CRTC1-MAML2 fusion in salivary glands…..7

Figure S7: Salivary gland tumors developed from the Line 2 mCre-CM(+) mice displayed a characteristic histological feature of human MEC and expressed the CRTC1-MAML2 fusion. ……………………………..8

Figure S8: Representative images showed abnormal ducts in the salivary glands of mCre-CM(+) mice...9

Figure S9: Expression levels of the CRTC1-MAML2 fusion and its known target genes in the CRTC1-MAML2 fusion-induced murine MEC tumors were analyzed by qRT-PCR 10

Figure S10: Enhanced expression of the top 10 genes shared by both the RB1/RBL1 loss-induced and CDK4/6-RB integrated signatures in mouse MEC tumors were valdiated by qRT-PCR……………………11

Figure S11: Immunohistochemical analysis of mouse MEC tumors for p-Rb, P16 and Cdk4.……………...12

Figure S12: Western blotting analysis of phosphorylated RB (p-RB) and total RB levels in human MEC H3118 cells with shRNA-mediated CRTC1-MAML2/MAML2 knockdown and with MAML2 specific Crispr-knockout. ……………………………………………………………………………………………………13

Figure S13: Combination index (CI) values of Palbociclib and Erlotinib treatment in human MEC colony assays were determined using CalcuSyn software…………………………………………………………14

Supplemental Tables (S1-S4)

Table S1: A list of genes show up-regulated expression in CRTC1-MAML2-induced murine MEC and down-regulated expression in CRTC1-MAML2-depleted human MEC cells or the reverse pattern... 15

Table S2: The top 15 enriched oncogenic signatures in the CRTC1-MAML2-induced murine MEC vs. normal salivary gland tissues are shown...19

Table S3: A list of differentially expressed genes in the CRTC1-MAML2-induced murine MEC show overlaps with the CDK4/6-RB signature and/or the RB1/RBL1 loss-induced gene set...20

Table S4: Primer sequences used for the qRT-PCR analysis in this study………………………….………24

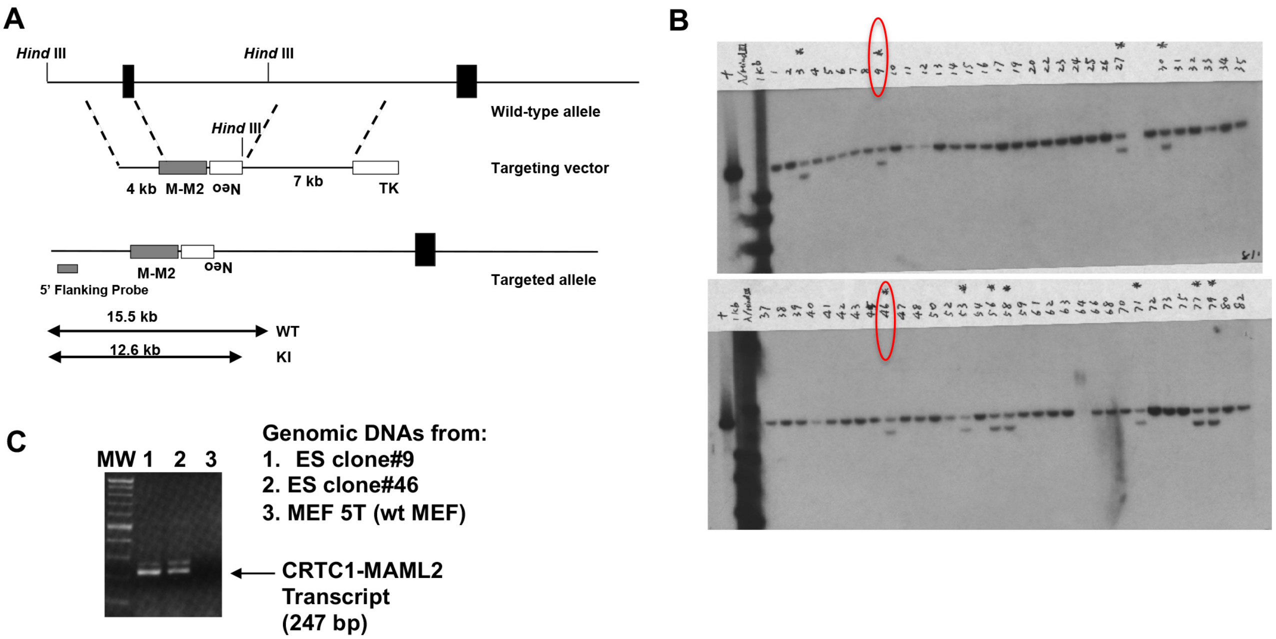

Figure S1: A mouse strain carrying a CRTC1-MAML2 knock-in allele under the *Crtc1* natural regulatory control (mimicking the t(11;19) genetic abnormality in human MEC) died after birth. (A) The CRTC1-MAML2 (M-M2) knock-in construct contained a 4 kb homologous arm (5' *Crtc1* upstream regulatory sequence) plus the CRTC1-MAML2 fusion open reading frame sequence, and a 7 kb 3' homologous arm that flanked the neo cassette in the pKOP906NeoTK-anti vector. **(B)** This construct was linearized with *Not*I and electroporated into Bruce 4 ES cells (derived from the C57BL/6 strain). The transfectant ES cells were selected in the presence of G418 and ganciclovir, and then expanded for Southern analysis. Specifically, the genomic DNA was isolated and digested with *Hind* III, and probed with a 5' flanking probe as shown in (A). The knock-in (KI) allele (giving rise to a 12.6 kb *Hind* III-digested fragment) was distinguished from the wildtype (WT) allele (producing a 15.5 kb fragment) by size. **(C)** Two ES clones (#9 and #46) were analyzed for the CRTC1-MAML2 transcripts by RT-PCR. The primer sequences were 5' CGGAAATTCAGCGAGAAGAT 3' (upper) and 5' GAGATTCTCCCCAACACGAA 3' (lower), which amplified a 247 bp fusion product. These two ES clones (#9, and #46) were thus validated for successful homologous recombination. They were subsequently used for the injections into Balb/C blastocysts, which were then transferred to recipient mothers (Swiss Webster mice) to generate chimeras as indicated by the presence of black coat color. A total of 13 Bl6/Balb/c chimera, 8 males & 5 females were obtained. Three of the mature chimeric males with the high percentage of black fur were then crossed with Balb/c females in order to produce germline founders. The pups that were positive for the fusion knock-in allele died immediately after birth.

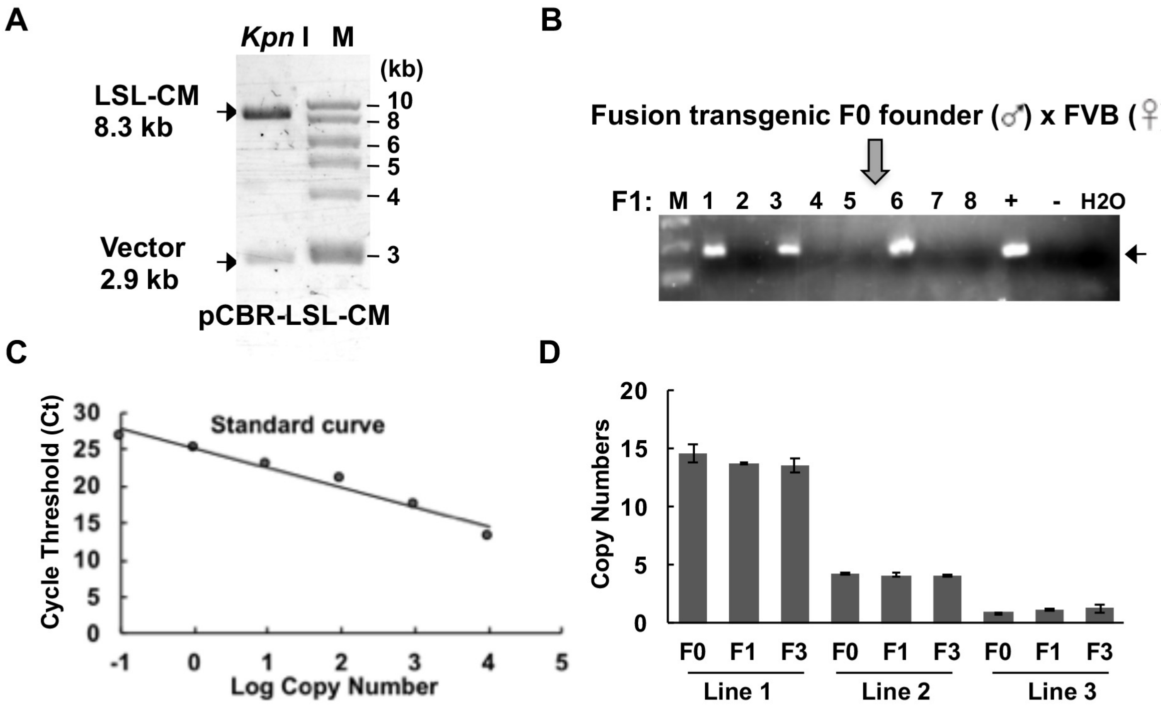

Figure S2: The transgene construct, genotyping, and transgene copy numbers in three Cre-regulated CRTC1-MAML2 transgenic mouse lines were shown. (A) The fusion transgene fragment LSL-CM (8.3kb) was isolated after the vector sequence (2.9 kb) was removed from the pCBR-LSL-CM construct with *Kpn*I digestion. The purified fusion transgenic fragments were used for microinjection into the pronuclei of FVB/J fertilized eggs to generate transgenic mice. **(B)** The F0 founder mice were bred to FVB mice and the transgene was detected in F1 offspring mice. PCR analysis of tail DNA of one litter of F1 offspring was shown. The pCBR-LSL-CM transgene construct were used as a positive control, and tail DNA from a FVB mouse and water were used as negative controls. **(C)** A standard curve was established to correlate cycle threshold (Ct value) with the transgene gene copy number of purified fusion transgene plasmids by qPCR. **(D)** The qPCR analysis of tail DNAs was performed to determine the transgene copy numbers for three transgenic lines based on the standard curve (C). Three transgenic lines maintained similar transgene copy numbers over three generations in comparison to their founder mice, indicating that the transgene is stably integrated into the mouse genome.

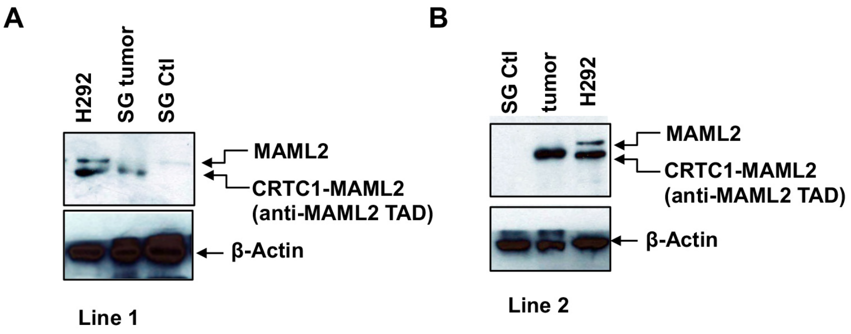

Figure S3: Western blotting analysis of the CRTC1-MAML2 fusion transgene expression in salivary gland tumors developed from Line 1 and Line 2 mCre-CM (+) mice. (A) Expression levels of the CRTC1-MAML2 transgenic gene in the salivary gland tumor developed from a Line 1 mCre-CM(+) mouse was analyzed. Salivary gland tissue from a transgene-negative mCre-CM(-) littermate was used as a negative control (SG Ctl) and human MEC H292 cells were used as positive control. Mouse β-Actin was blotted as a loading control. **(B**) A similar analysis was conducted on the salivary gland tumors developed from a Line 2 mCre-CM(+) mouse.

.

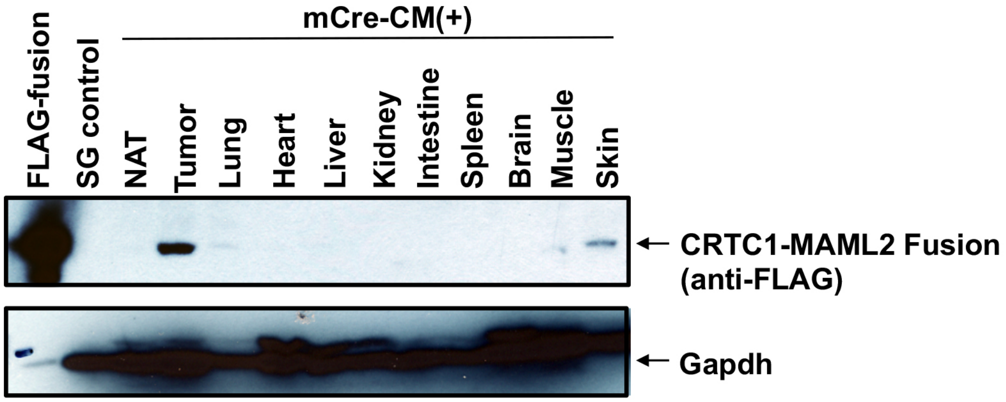

Figure S4: Western blot analysis of the CRTC1-MAML2 transgene expression in various tissues of the mCre;CM(+) mouse line. Western blot analysis was performed to determine the CRTC1-MAML2 transgene expression in a salivary gland tumor (T) and its adjacent normal tissue (NAT) and various tissues collected from a mCre-CM1(+) mouse (Line 1). The salivary gland tissue from an age-matched transgene-negative mCre-CM(-) mouse was use as a negative control (SG Control). Protein lysates prepared from 293T cells expressing FLAG-tagged CRTC1-MAML2 fusion were used as a positive control (FLAG-fusion). The transgenic FLAG-tagged CRTC1-MAML2 fusion protein was detected by anti-FLAG antibodies. Mouse Gapdh was blotted as a loading control.

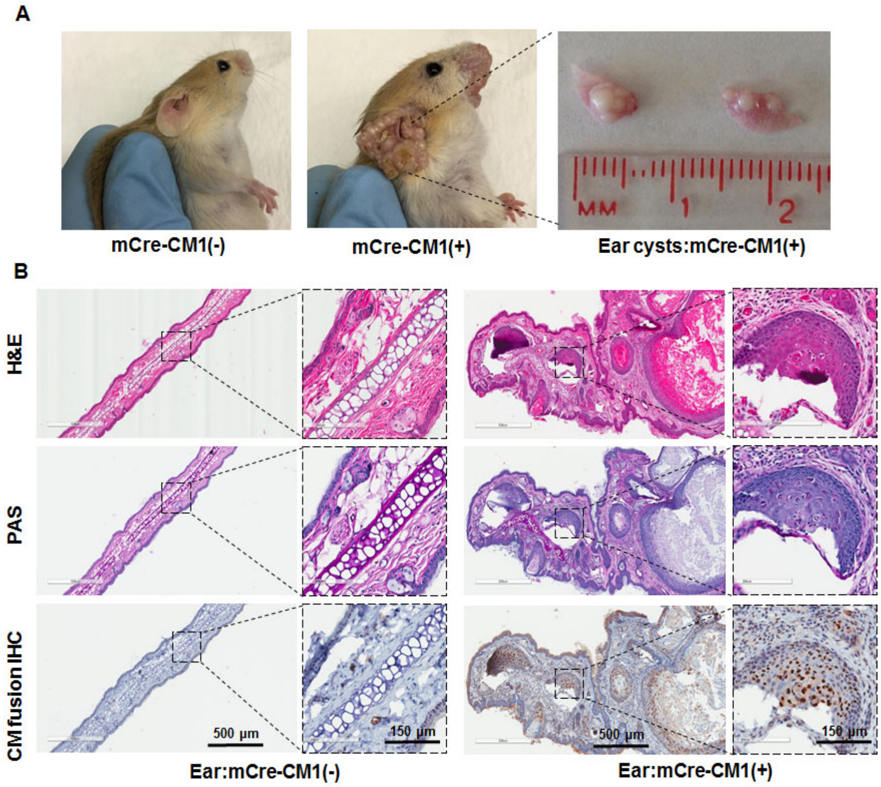

Figure S5: The mCre-CM1(+) mice developed ear cysts with CRTC1-MAML2 fusion expression. (A) Representative images show a mCre-CM (+) mouse (Line 1) with ear cysts and a transgene-negative mCre-CM(-) mouse control. **(B)** The ear tissues of mCre-CM (-) and mCre-CM (+) mice were stained with H&E for histological analysis, PAS to detect mucin-expressing cells, and IHC with MAML2 TAD antibodies to detect the transgenic CRTC1-MAML2 fusion expression. Scale bar: 500 µm (left panels), 150 µm (right panels)

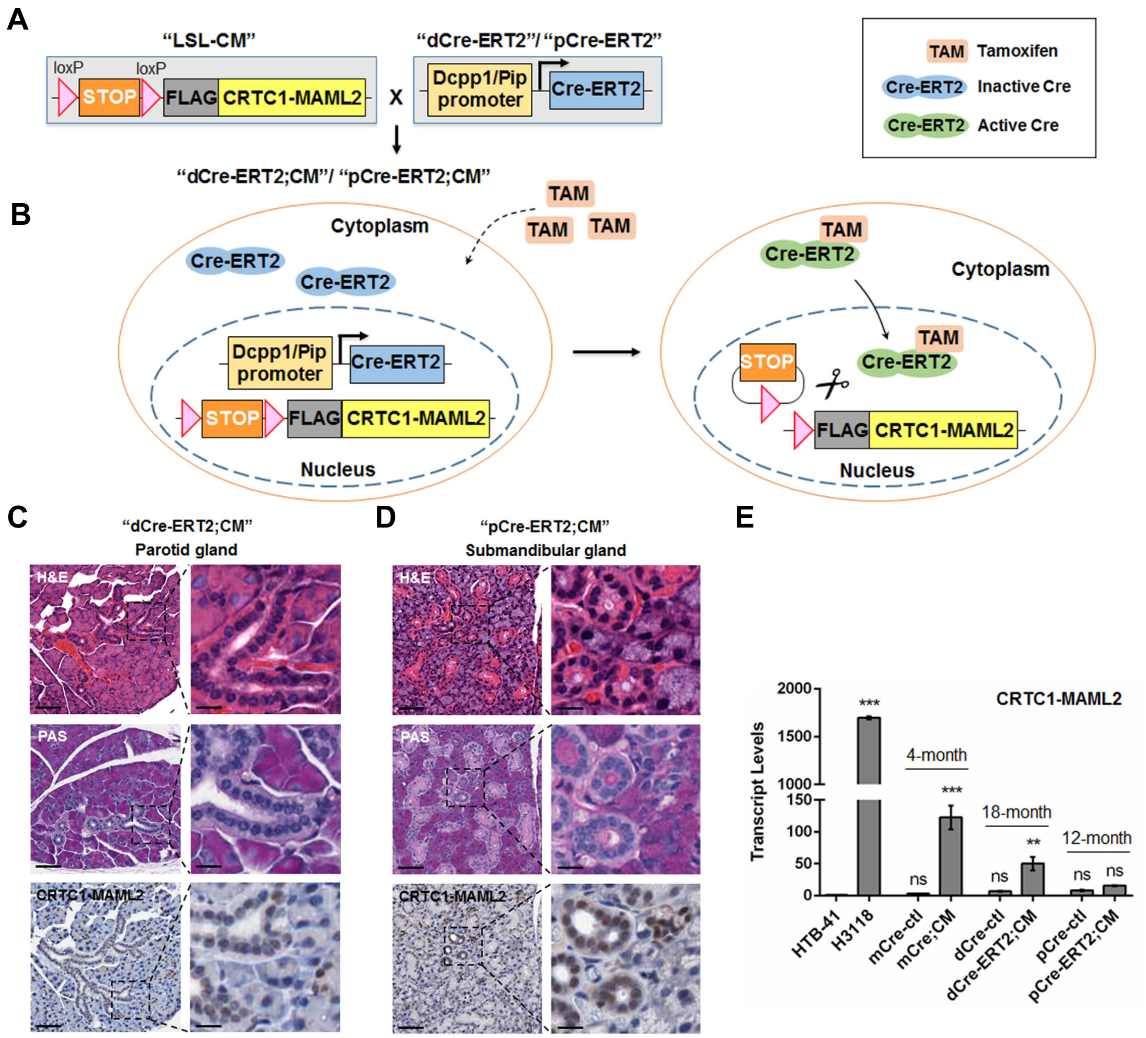

Figure S6: The Cre-regulated CRTC1-MAML2 transgenic line, after being crossed with Dcpp1-Cre-ERT2 or Pip-Cre-ERT2 transgenic mice, expressed low levels of the CRTC1-MAML2 fusion in salivary glands. **(A)** Breeding strategies for obtaining "dCre-ERT2; CM" and "pCre-ERT2; CM" mice were shown. **(B)** A schematic diagram showed that tamoxifen (TAM)-induced Cre recombination leads to the expression of the CRTC1-MAML2 fusion transgene. **(C)** The "dCre-ERT2; CM" mice were injected with TAM at about 4 weeks old for 3 consecutive days. The images of HE staining, PAS staining and the CRTC1-MAML2 IHC of parotid glands of a representative "dCre-ERT2; CM" mouse (male, 18-month old) were shown. **(D)** The "pCre-ERT2; CM" mice were injected with TAM at about 4 weeks old for 3 consecutive days. The images of HE staining, PAS staining and the CRTC1-MAML2 IHC of submandibular glands of a representative "pCre-ERT2; CM" mouse (female, 12-month old) were shown. Scale bar:100 μm (left panels), 25 μm (right panels). **(E)** The transcript levels of the CRTC1-MAML2 fusion transgene in salivary glands of "mCre; CM", "dCre-ERT2; CM" and "pCre-ERT2; CM" mice were determined by qRT-PCR. HTB-41 and H3118 were used as negative and positive controls for the CRTC1-MAML2 fusion, respectively. "mCre-ctl", "dCre-ctl" and "pCre-ctl" were age-matched controls containing Cre but without the CRTC1-MAML2 fusion transgene. One-way ANOVA was used for multiple comparisons (**p<0.01, *** p<0.001).

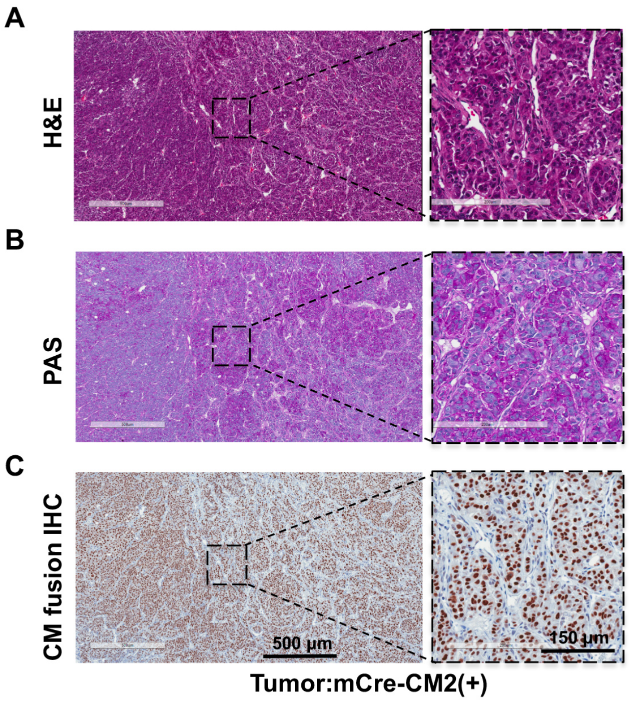

Figure S7: Salivary gland tumors developed from the Line 2 mCre-CM(+) mice displayed some histological features of human MEC and expressed the CRTC1-MAML2 fusion. Salivary gland tumor developed from a mCre-CM(+) mouse (generated from the mating of Cre-regulated CRTC1-MAML2 mouse line 2 with the mCre line) was subjected to H&E staining **(A)**; PAS staining for detecting mucin-expressing cells **(B)**; and IHC staining with anti-MAML2 TAD antibodies for detecting the transgenic CRTC1-MAML2 fusion expression **(C)**. Scale bar: 500 µm (left panels), 150 µm (right panels).

A B

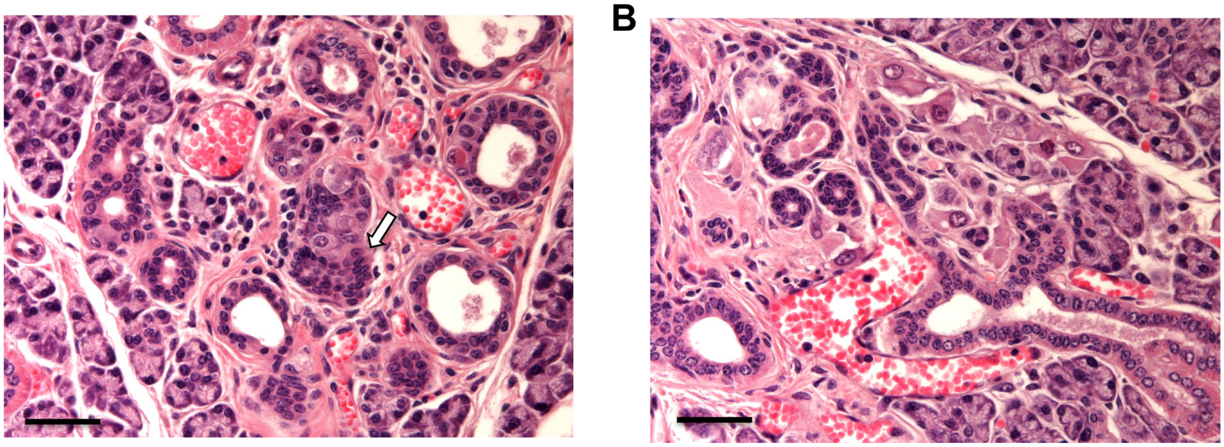

Figure S8: Representative images showed abnormal ducts in the salivary glands of mCre-CM(+) mice (without palpable tumors). Sections of submandibular glands from two mCre-CM(+) mice of around 10 weeks old were stained by H&E **(A, B)**. Abnormal cells within a duct were marked by a white arrow (A). Scale bars: 50 μm.

.

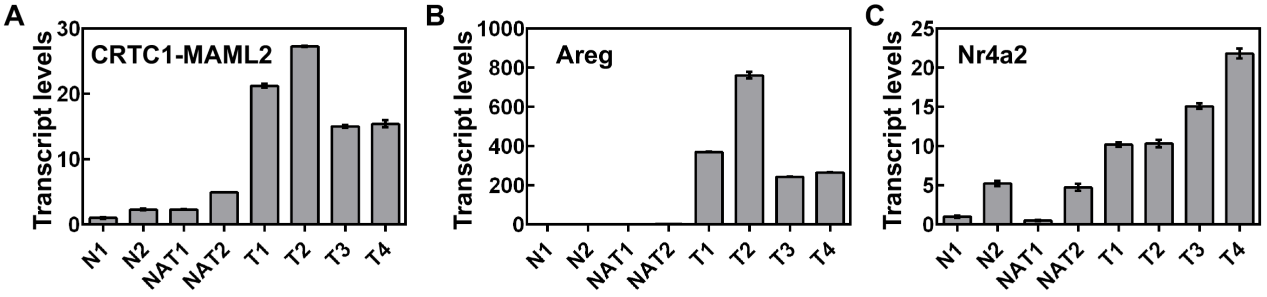

Figure S9: Expression levels of the CRTC1-MAML2 fusion and its known target genes in the CRTC1-MAML2 fusion-induced murine MEC tumors were analyzed by qRT-PCR. The CRTC1-MAML2 fusion-induced salivary gland tumors (T1, T2), their matched tumor adjacent normal tissues (NAT1, NAT2), normal salivary gland tissues from the control non-transgene carrier littermates (N1, N2), and also 2 salivary gland tumors from separate mice (T3, T4) were used in this analysis. **(A)** The CRTC1-MAML2 fusion transgene was highly expressed in tumors but low or undetectable expressed in tumor adjacent normal tissues and normal salivary gland tissues. **(B, C)** The CRTC1-MAML2 target genes Areg (B) and Nr4a2 (C) were significantly upregulated in tumors. The CRTC1-MAML2, Areg and Nr4a2 mRNA expressions were normalized to the levels of reference gene Gapdh.

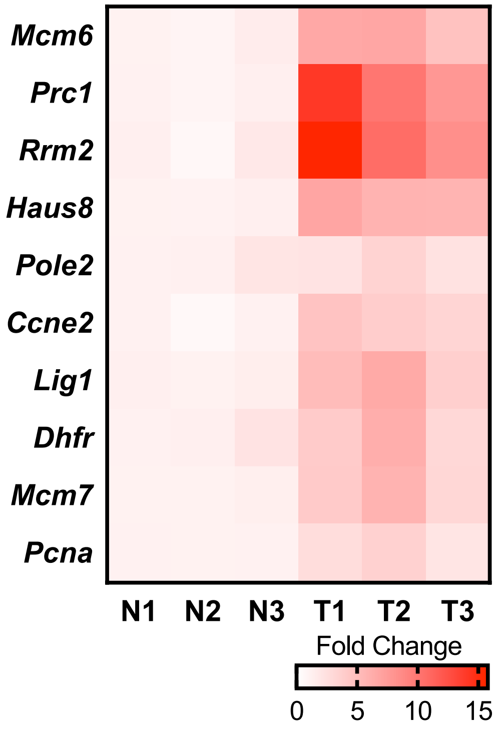

Figure S10: Enhanced expression of the top 10 genes shared by both the RB1/RBL1 loss-induced and CDK4/6-RB integrated signatures in mouse MEC tumors were validated by qRT-PCR. RNAs isolated from 3 salivary gland tumors from the mCre-CM mice and 3 normal salivary gland tissues from the control littermates were used for qRT-PCR analysis, and the expression was normalized using the control *Hprt* gene. The relative expression data were represented as a heatmap using Prism GraphPad 8.

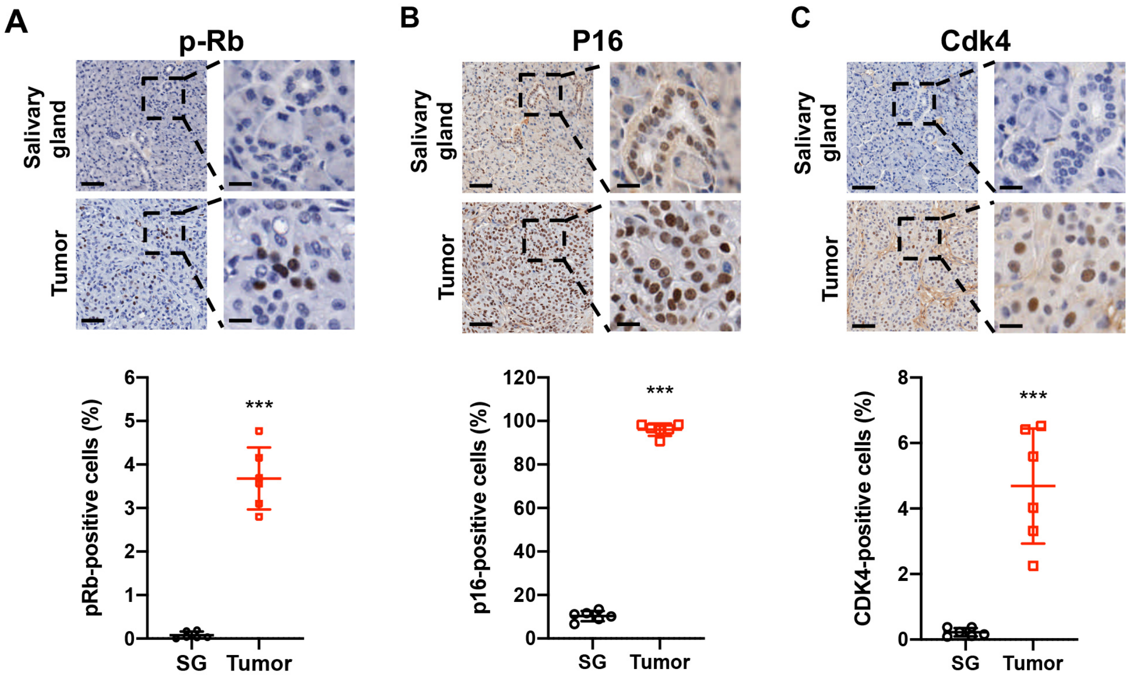

Figure S11: Immunohistochemical analysis of mouse MEC tumors for p-Rb, P16 and Cdk4. Paraffin-embedded mouse MEC tumors and normal salivary gland tissues from the non-transgene carrier control mice were subjected to IHC staining using phospho-Rb (Ser807/811) (Cell signaling # 8516), p16 (Santa Cruz # sc-1661), and Cdk4 (Santa Cruz # sc-166373). Representatives of IHC staining for **(A)** p-Rb, **(B)** p16 and **(C)** Cdk4 were shown (left bar = 100 µm, right bar = 25 µm). The positively-stained cells were quantified in 6 randomly selected visual fields (5x, 1000x1000 pixels) using ImageJ. The result was presented as percentage of positively-stained cells per visual field. Two-tailed t-test was used to calculate the p values (***p<0.001).

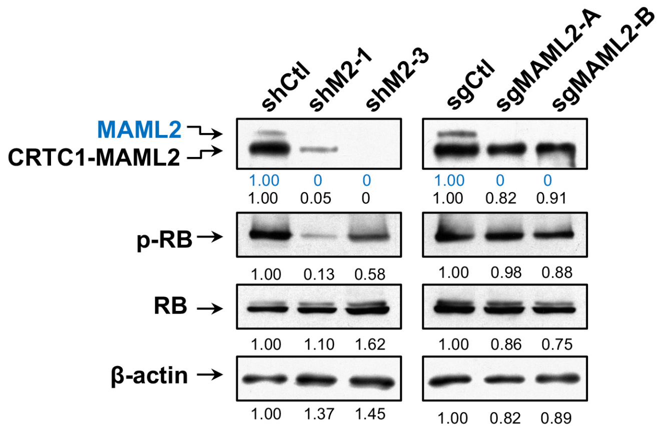

Figure S12: Western blotting analysis of phosphorylated RB (p-RB) and total RB levels in human MEC H3118 cells with shRNA-mediated CRTC1-MAML2/MAML2 knockdown and with CRISPR/Cas9-mediated MAML2 knockout. Human MEC H3118 cells were transduced with lentiviruses expressing shRNAs (shM2-1, 5'-CCCTGTCTAAACTCCAGGATA-3'; shM2-3, 5'-CCCAAAGCAATTGTTAGCAAA-3') that target the respective 3' UTR and TAD domain of the CRTC1-MAML2 fusion and MAML2, or scramble shRNA control (Ctl). Cell lysates were prepared at 96 hours after viral infection for Western blotting (left panel). Stable Cas9-expressing H3118 cells were transduced with lentiviruses expressing sgRNAs that target the exon 1 of MAML2 (sgMAML2-A, 5'-TGTGAAGGACGATATGAACG-3'; sgMAML2-B, 5'-GATAGCACTGTGCACTCTCG-3') or control (sgCtl), and cell lysates were prepared at 96 hours after viral infection for Western blotting (right panel). Western blotting was performed using antibodies against phospho-RB (Ser807/811) (CST #8516) and RB (CST554136), and β-actin (Sigma #5136). The phospho-RB (Ser807/811) level was reduced in CRTC1-MAML2/MAML2 knockdown but relatively unchanged in CRISPR/Cas9-mediated MAML2 knockout H3118 cells, whereas the total RB level remained at similar levels, indicating that the depletion of fusion reduced phosphorylated RB level. β-ACTIN was used as a loading control.

Cell line	ED50	ED75	ED90
H3118	0.164	0.184	0.206
HMC-3B	< 0.001	< 0.001	< 0.001
H292	0.790	0.184	0.087

◆ Strong synergism (CI<0.3)

◆ Moderate synergism (CI=0.3-0.85)

Figure S13: Combination index (CI) values of palbociclib and erlotinib treatment in human MEC colony assays were determined using CalcuSyn software. CI<1, CI=1, and CI>1 indicate synergism, additivity and antagonism, respectively.

Table S1: A list of genes show up-regulated expression in CRTC1-MAML2-induced murine MEC and down-regulated expression in CRTC1-MAML2-depleted human MEC cells or the reverse pattern. *Differential expressed genes (DEGs) in mouse MEC vs normal salivary gland tissues were defined using cut-off criteria of absolute fold change of >=2.0 and FDR p value of =< 0.05. These genes were compared with the reported DEGs in fusion-depleted human MEC H3118 cells vs control cells (Chen et al., BMC Cancer 2015). Red: upregulated; blue: down-regulated.

ProbeName	Gene Name	Gene description	Fold Change (Tumor/Normal)	FDR p-value	probeID	Gene Symbol	Fold change (fusion-depleted vs control H3118)	p-value
ASMM10P010373	**Pcsk1**	proprotein convertase subtilisin/kexin type 1	1976.87	4.77E-05	205825_at	PCSK1	-5.7	0.00048
ASMM10P022539	**Ptgs2**	prostaglandin-endoperoxide synthase 2	575.31	4.14E-05	1554997_a_a	PTGS2	-2.41	0.02817
ASMM10P031996	**Areg**	amphiregulin	512.99	5.58E-05	205239_at	AREG	-2.23	0.04495
ASMM10P012140	**Stc1**	stanniocalcin 1	344.87	4.77E-05	204597_x_at	STC1	-21.74	0
ASMM10P005076	**Smoc1**	SPARC related modular calcium binding 1	287.14	0.0001065	222783_s_at	SMOC1	-6.47	0
ASMM10P034033	**Pthlh**	parathyroid hormone-like peptide	207.66	0.0001281	206300_s_at	PTHLH	-3.82	0.00035
ASMM10P036818	**Calca**	calcitonin/calcitonin-related polypeptide, alpha	175.81	4.14E-05	217561_at	CALCA	-3.49	0.00643
ASMM10P027766	**Mfsd2a**	major facilitator superfamily domain containing 2A	127.35	8.16E-05	225316_at	MFSD2A	-4.96	5.00E-05
ASMM10P021239	**Ano3**	anoctamin 3	85.62	0.0004157	215241_at	ANO3	-2.5	0.00317
ASMM10P028730	**Car9**	carbonic anhydrase 9	78.25	0.0002255	205199_at	CA9	-2.77	0.00402
ASMM10P021291	**Grem1**	gremlin 1	57.19	0.0002212	218469_at	GREM1	-5.21	0.00105
ASMM10P005824	**Smox**	spermine oxidase	56.33	0.0001664	210357_s_at	SMOX	-2.95	0.01324
ASMM10P041830	**C2cd4a**	C2 calcium-dependent domain containing 4A	51.50	0.0011874	241031_at	C2CD4A	-7.84	0.00011
ASMM10P051974	**Ube2ql1**	ubiquitin-conjugating enzyme E2Q family-like 1	46.96	0.0029308	226612_at	UBE2QL1	-6.81	0
ASMM10P040244	**Sfrp1**	secreted frizzled-related protein 1	35.46	0.0007672	202037_s_at	SFRP1	-3.12	0.00018
ASMM10P044579	**Il13ra2**	interleukin 13 receptor, alpha 2	33.74	0.0013247	206172_at	IL13RA2	-2.55	0.01144
ASMM10P006754	**Nid2**	nidogen 2	29.81	0.0002013	204114_at	NID2	-2.49	0.00011
ASMM10P020398	**Ptges**	prostaglandin E synthase	28.56	7.37E-05	210367_s_at	PTGES	-2.81	0.0132
ASMM10P048901	**Cd55**	CD55 antigen	25.09	0.0023295	201925_s_at	CD55	-2.37	1.00E-04
ASMM10P011052	**Grem2**	gremlin 2 homolog, cysteine knot superfamily (Xenopus laevis)	23.01	0.0012192	240509_s_at	GREM2	-3.95	0.00271
ASMM10P040321	**Dusp4**	dual specificity phosphatase 4	22.24	0.0005955	204015_s_at	DUSP4	-3.93	0.00155
ASMM10P007618	**Tnfrsf10b**	tumor necrosis factor receptor superfamily, member 10b	21.97	8.82E-05	210405_x_at	TNFRSF10B	-2.89	0.00018
ASMM10P032138	**Ephx4**	epoxide hydrolase 4	21.72	0.0133852	239579_at	EPHX4	-2.15	0.00106
ASMM10P010572	**Pde4d**	phosphodiesterase 4D, cAMP specific	19.74	0.0001943	228962_at	PDE4D	-2.93	0.00034
ASMM10P009666	**Raet1e**	retinoic acid early transcript 1E	18.48	0.0005421	1552777_a_a	RAET1E	-2.72	0.02536
ASMM10P051632	**Pxdc1**	PX domain containing 1	17.38	0.004974	231387_at	PXDC1	-2.16	0.00584
ASMM10P019230	**Avpi1**	arginine vasopressin-induced 1	16.73	0.000244	218631_at	AVPI1	-2.47	0.00015
ASMM10P015787	**Dusp1**	dual specificity phosphatase 1	16.33	0.002046	201041_s_at	DUSP1	-4.81	0.00017
ASMM10P029713	**Fuca1**	fucosidase, alpha-L- 1, tissue	16.12	0.0014108	202838_at	FUCA1	-3.09	0.0048
ASMM10P032468	**Bri3bp**	Bri3 binding protein	16.05	0.0018799	231810_at	BRI3BP	-2.15	0.0253
ASMM10P025736	**Usp13**	ubiquitin specific peptidase 13 (isopeptidase T-3)	16.00	0.0017911	205356_at	USP13	-2.8	0.00813
ASMM10P042737	**Mcam**	melanoma cell adhesion molecule	11.26	0.000627	1568191_at	MCAM	-3.45	7.00E-05
ASMM10P011237	**Ptp4a1**	protein tyrosine phosphatase 4a1	10.23	0.0017695	200730_s_at	PTP4A1	-2.12	0.00068
ASMM10P008715	**Dst**	dystonin	10.06	0.0014659	232098_at	DST	-2.11	0.03694
ASMM10P050921	**Snapc1**	small nuclear RNA activating complex, polypeptide 1	9.37	0.0001815	205443_at	SNAPC1	-2.01	0.00323
ASMM10P015058	**Bdh1**	3-hydroxybutyrate dehydrogenase, type 1	9.09	0.0042887	211715_s_at	BDH1	-2.25	0.00152
ASMM10P018767	**Aldh3b1**	aldehyde dehydrogenase 3 family, member B1	8.87	0.0012238	205640_at	ALDH3B1	-2.48	9.00E-05
ASMM10P006778	**Pecam1**	platelet/endothelial cell adhesion molecule 1	8.73	0.0406114	208982_at	PECAM1	-6.92	0
ASMM10P055057	**Fkbp7**	FK506 binding protein 7	8.36	0.0004403	224002_s_at	FKBP7	-2.13	0.00893
ASMM10P050653	**Odc1**	ornithine decarboxylase, structural 1	8.32	0.0007386	200790_at	ODC1	-2.54	0.0019

ASMM10P055024	**Kynu**	kynureninase (L-kynurenine hydrolase)	8.32	0.0197558	210663_s_at	KYNU	-2.63	0.00098
ASMM10P022765	**Arpc5l**	actin related protein 2/3 complex, subunit 5-like	8.05	0.0076867	226914_at	ARPC5L	-2.23	0.00244
ASMM10P018786	**Syt12**	synaptotagmin XII	7.89	0.0008355	228072_at	SYT12	-2.97	0.00015
ASMM10P000286	**Mprip**	myosin phosphatase Rho interacting protein	7.45	0.0019086	214694_at	MPRIP	-2.54	0.00113
ASMM10P018788	**2010003K11I**	RIKEN cDNA 2010003K11 gene	7.33	0.0013587	233938_at	C11orf86	-2.31	0.00125
ASMM10P039328	**Snx25**	sorting nexin 25	7.30	0.000166	232425_at	SNX25	-2.36	0.01993
ASMM10P045191	**Hal**	histidine ammonia lyase	6.40	0.013382	206643_at	HAL	-6.79	2.00E-05
ASMM10P031434	**Steap4**	STEAP family member 4	6.14	0.0207594	225987_at	STEAP4	-3.03	0.00022
ASMM10P045625	**Lum**	lumican	6.14	0.0027542	201744_s_at	LUM	-6.98	0.00061
ASMM10P039750	**Cdh11**	cadherin 11	5.62	0.0015414	207173_x_at	CDH11	-3.38	0.00044
ASMM10P029844	**Casp9**	caspase 9	5.51	0.0005406	210775_x_at	CASP9	-2.33	0.00016
ASMM10P016441	**Xdh**	xanthine dehydrogenase	5.50	0.0016017	210301_at	XDH	-2.87	0.02018
ASMM10P052596	**Serpinb9**	serine (or cysteine) peptidase inhibitor, clade B, member 9	5.32	0.0379964	242814_at	SERPINB9	-2.98	0.00472
ASMM10P048403	**Havcr2**	hepatitis A virus cellular receptor 2	5.29	0.0003409	235458_at	HAVCR2	-3.16	5.00E-05
ASMM10P031029	**Fam216a**	family with sequence similarity 216, member A	5.11	0.0009493	204521_at	FAM216A	-2.16	0.00016
ASMM10P042038	**Zbtb38**	zinc finger and BTB domain containing 38	5.09	0.0086691	219221_at	ZBTB38	-2.19	0.04405
ASMM10P015055	**Fam43a**	family with sequence similarity 43, member A	5.01	0.0053646	227410_at	FAM43A	-2.47	0.00646
ASMM10P039881	**Tmem231**	transmembrane protein 231	4.77	0.0011815	64900_at	TMEM231	-2.03	0.02144
ASMM10P019387	**Gfra1**	glial cell line derived neurotrophic factor family receptor alpha 1	4.71	0.0018261	227550_at	GFRA1	-4.56	0.00966
ASMM10P030279	**Srgn**	serglycin	4.62	0.0217835	201859_at	SRGN	-3.95	0.00518
ASMM10P055466	**Sh3gl2**	SH3-domain GRB2-like 2	4.44	0.0150867	205751_at	SH3GL2	-2.15	0.00032
ASMM10P022699	**Ptgs1**	prostaglandin-endoperoxide synthase 1	4.42	0.0054977	205128_x_at	PTGS1	-3.89	0
ASMM10P050949	**Arg2**	arginase type II	4.40	0.0206531	203946_s_at	ARG2	-7.79	5.00E-05
ASMM10P042080	**Slc35g2**	solute carrier family 35, member G2	4.40	0.0231678	219569_s_at	SLC35G2	-2.57	0.00077
ASMM10P054381	**Ednrb**	endothelin receptor type B	4.26	0.0069715	204271_s_at	EDNRB	-2.23	0.00037
ASMM10P009532	**Sik2**	salt inducible kinase 2	3.95	0.0035941	223430_at	SIK2	-4.06	0
ASMM10P008574	**Il6**	interleukin 6	3.95	0.0435905	205207_at	IL6	-3.35	0.00259
ASMM10P042706	**Clmp**	CXADR-like membrane protein	3.56	0.001889	228082_at	CLMP	-2.63	7.00E-05
ASMM10P051910	**Zfp367**	zinc finger protein 367	3.45	0.0050865	229551_x_at	ZNF367	-2.31	0.00111
ASMM10P050919	**Prkch**	protein kinase C, eta	3.40	0.0304536	218764_at	PRKCH	-2.16	0.00061
ASMM10P007977	**Ergic1**	endoplasmic reticulum-golgi intermediate compartment (ERGIC	3.39	0.0057481	223847_s_at	ERGIC1	-2.01	0.00621
ASMM10P016623	**Pde10a**	phosphodiesterase 10A	3.35	0.009019	205501_at	PDE10A	-3.32	1.00E-04
ASMM10P011302	**Fndc3a**	fibronectin type III domain containing 3A	3.28	0.0288606	238961_s_at	FNDC3A	-2.09	0.04589
ASMM10P027567	**Aig1**	androgen-induced 1	3.07	0.0229018	230520_at	AIG1	-3.42	1.00E-05
ASMM10P053678	**Zc3h12b**	zinc finger CCCH-type containing 12B	2.92	0.0107874	229234_at	ZC3H12B	-2.23	0.00143
ASMM10P009234	**Tango6**	transport and golgi organization 6	2.90	0.0054289	226467_at	TMCO7	-3.54	7.00E-05
ASMM10P006004	**C1qtnf6**	C1q and tumor necrosis factor related protein 6	2.88	0.0024489	223571_at	C1QTNF6	-2.81	0.00023
ASMM10P008520	**Pigh**	phosphatidylinositol glycan anchor biosynthesis, class H	2.83	0.0021492	209625_at	PIGH	-2.5	0.00017
ASMM10P044186	**Pdk3**	pyruvate dehydrogenase kinase, isoenzyme 3	2.79	0.0018319	230085_at	PDK3	-2.4	0.03499
ASMM10P031711	**Lyar**	Ly1 antibody reactive clone	2.79	0.0228302	223413_s_at	LYAR	-2.01	0.00091
ASMM10P006636	**Daf2**	decay accelerating factor 2	2.58	0.0406508	201925_s_at	CD55	-2.37	1.00E-04
ASMM10P026916	**B4galt1**	UDP-Gal:betaGlcNAc beta 1,4- galactosyltransferase, polypeptid	2.36	0.0248671	229403_at	B4GALT1	-2.21	0.0161
ASMM10P000414	**Slc8a1**	solute carrier family 8 (sodium/calcium exchanger), member 1	2.35	0.0067599	235518_at	SLC8A1	-9.25	0.00011
ASMM10P042070	**Cldn18**	claudin 18	2.24	0.0407458	232578_at	CLDN18	-2.76	0.00058
ASMM10P055093	**Gm13889**	predicted gene 13889	2.19	0.0080369	227099_s_at	C11orf96	-2.03	0.01409
ASMM10P053322	**4933411K20I**	RIKEN cDNA 4933411K20 gene	2.04	0.0389594	230567_at	KIAA1430	-2.17	0.0074
ASMM10P030243	**Tyms**	thymidylate synthase	2.02	0.0466357	1554696_s_a	TYMS	-2.1	0.02143

ASMM10P046718	**Fat2**	FAT tumor suppressor homolog 2 (Drosophila)	-2.02	0.0363304	208153_s_at	FAT2	2.01	0.02442
ASMM10P039033	**Frk**	fyn-related kinase	-2.02	0.0227679	235924_at	N73742	2.31	0.00379
ASMM10P017925	**Ablim3**	actin binding LIM protein family, member 3	-2.05	0.031373	205730_s_at	ABLIM3	4.23	0.00021
ASMM10P007946	**Pdzk1ip1**	PDZK1 interacting protein 1	-2.05	0.0169408	1553589_a_a	PDZK1IP1	2.02	0.01489
ASMM10P053126	**Tead2**	TEA domain family member 2	-2.06	0.0200521	226408_at	TEAD2	2.61	0.01421
ASMM10P047175	**Ngef**	neuronal guanine nucleotide exchange factor	-2.08	0.0136808	227240_at	NGEF	2.08	0.00323
ASMM10P018456	**Sh3rf2**	SH3 domain containing ring finger 2	-2.09	0.0218509	243582_at	SH3RF2	3.83	2.00E-05
ASMM10P046827	**Slc47a2**	solute carrier family 47, member 2	-2.12	0.0468783	231068_at	SLC47A2	3.61	0.00311
ASMM10P009334	**Nmnat2**	nicotinamide nucleotide adenylyltransferase 2	-2.16	0.0220768	1552712_a_a	NMNAT2	2.41	0.01638
ASMM10P033990	**St8sia1**	ST8 alpha-N-acetyl-neuraminide alpha-2,8-sialyltransferase 1	-2.18	0.045462	210073_at	ST8SIA1	2.28	0.00262
ASMM10P004992	**Bean1**	brain expressed, associated with Nedd4, 1	-2.20	0.0057153	214068_at	BEAN1	2.14	0.00478
ASMM10P042893	**Stra6**	stimulated by retinoic acid gene 6	-2.25	0.0196125	221701_s_at	STRA6	2.66	0.00108
ASMM10P034463	**Gpnmb**	glycoprotein (transmembrane) nmb	-2.30	0.0123661	1554018_at	GPNMB	16	2.00E-05
ASMM10P008527	**Gpr115**	G protein-coupled receptor 115	-2.30	0.0076672	237690_at	GPR115	3.48	1.00E-04
ASMM10P007338	**Tnf**	tumor necrosis factor	-2.37	0.0051632	207113_s_at	TNF	2.46	0.00053
ASMM10P013411	**Rims2**	regulating synaptic membrane exocytosis 2	-2.43	0.0201346	229823_at	RIMS2	3.15	0.00195
ASMM10P020692	**Scn3a**	sodium channel, voltage-gated, type III, alpha	-2.43	0.0104088	210432_s_at	SCN3A	2.84	0.02159
ASMM10P016962	**Pim1**	proviral integration site 1	-2.49	0.0450781	209193_at	PIM1	3.29	1.00E-05
ASMM10P052004	**Fmo4**	flavin containing monooxygenase 4	-2.53	0.0136071	206263_at	FMO4	2.28	0.00582
ASMM10P026413	**Vav3**	vav 3 oncogene	-2.71	0.0076537	218807_at	VAV3	7.17	0.00034
ASMM10P014728	**Runx1**	runt related transcription factor 1	-2.87	0.0208811	210365_at	RUNX1	2.3	0.00957
ASMM10P043173	**Ctsh**	cathepsin H	-2.94	0.0107126	202295_s_at	CTSH	3.27	9.00E-05
ASMM10P055664	**Hs3st1**	heparan sulfate (glucosamine) 3-O-sulfotransferase 1	-3.07	0.0017335	205466_s_at	HS3ST1	2.03	0.02889
ASMM10P012590	**Mtss1**	metastasis suppressor 1	-3.14	0.001303	203037_s_at	MTSS1	4.83	0.00047
ASMM10P003034	**Mx1**	myxovirus (influenza virus) resistance 1	-3.33	0.0024641	202086_at	MX1	2.34	0.00665
ASMM10P043171	**Rasgrf1**	RAS protein-specific guanine nucleotide-releasing factor 1	-3.33	0.0014739	210550_s_at	RASGRF1	3.88	4.00E-05
ASMM10P013160	**Krt75**	keratin 75	-3.34	0.0046238	207065_at	KRT75	4.93	0.00057
ASMM10P038704	**Hs3st2**	heparan sulfate (glucosamine) 3-O-sulfotransferase 2	-3.38	0.004126	219697_at	HS3ST2	2.64	0.00102
ASMM10P053679	**Chil1**	chitinase-like 1	-3.46	0.0027211	209395_at	CHI3L1	5.08	0.00545
ASMM10P005813	**Cd44**	CD44 antigen	-3.53	0.0017569	217523_at	CD44	2.62	0.00099
ASMM10P054918	**Loxl4**	lysyl oxidase-like 4	-3.67	0.0047882	227145_at	LOXL4	2.63	0.00381
ASMM10P029984	**Hes2**	hairy and enhancer of split 2 (Drosophila)	-3.74	0.0108919	231928_at	HES2	3.67	0.01278
ASMM10P024849	**Gria2**	glutamate receptor, ionotropic, AMPA2 (alpha 2)	-3.77	0.0017491	205358_at	GRIA2	2.06	0.00059
ASMM10P017820	**Jakmip2**	janus kinase and microtubule interacting protein 2	-3.87	0.0238818	205888_s_at	JAKMIP2	2.05	0.01565
ASMM10P019821	**Ifit3**	interferon-induced protein with tetratricopeptide repeats 3	-3.87	0.0012731	229450_at	IFIT3	3.45	2.00E-05
ASMM10P050390	**Serpina1a**	serine (or cysteine) peptidase inhibitor, clade A, member 1A	-3.92	0.0011933	230318_at	SERPINA1	2.27	0.00614
ASMM10P017859	**Lox**	lysyl oxidase	-3.94	0.0394524	215446_s_at	LOX	2.23	0.00388
ASMM10P018571	**Csf1r**	colony stimulating factor 1 receptor	-4.03	0.0034883	203104_at	CSF1R	2.63	0.00231
ASMM10P011679	**Il17rd**	interleukin 17 receptor D	-4.23	0.0031068	227997_at	IL17RD	2.01	0.00123
ASMM10P022899	**Scn2a1**	sodium channel, voltage-gated, type II, alpha 1	-4.59	0.0048393	229057_at	SCN2A	2.56	0.04753
ASMM10P009394	**Ifitm10**	interferon induced transmembrane protein 10	-5.17	0.0033721	227863_at	IFITM10	2.38	0.04561
ASMM10P037845	**Klk6**	kallikrein related-peptidase 6	-5.20	0.0061439	204733_at	KLK6	3.98	2.00E-05
ASMM10P029173	**Ror1**	receptor tyrosine kinase-like orphan receptor 1	-5.35	0.0011487	232060_at	ROR1	2.03	0.0211
ASMM10P002415	**L1cam**	L1 cell adhesion molecule	-5.38	0.0005547	204584_at	L1CAM	2.75	0.01583
ASMM10P042698	**Scn3b**	sodium channel, voltage-gated, type III, beta	-5.51	0.0018236	204723_at	SCN3B	2.52	0.00298
ASMM10P052346	**Akr1c21**	aldo-keto reductase family 1, member C21	-5.68	0.0032528	217626_at	AKR1C1	3	0.00137
ASMM10P025788	**Dusp10**	dual specificity phosphatase 10	-5.91	0.001261	215501_s_at	DUSP10	2.33	0.00032

ASMM10P034924	**Lrrn1**	leucine rich repeat protein 1, neuronal	-5.93	0.0062193	226884_at	LRRN1	2.79	8.00E-05
ASMM10P053862	**Grik2**	glutamate receptor, ionotropic, kainate 2 (beta 2)	-5.93	0.0043846	213845_at	GRIK2	2.05	0.00074
ASMM10P020839	**AA986860**	expressed sequence AA986860	-5.96	0.000341	219476_at	C1orf116	2.47	0.00197
ASMM10P032914	**Gpr37**	G protein-coupled receptor 37	-6.26	0.0329451	209631_s_at	GPR37	2.05	0.02429
ASMM10P052209	**Atp1b1**	ATPase, Na+/K+ transporting, beta 1 polypeptide	-6.57	0.0017782	201242_s_at	ATP1B1	2.09	0.00895
ASMM10P029019	**Lurap1l**	leucine rich adaptor protein 1-like	-7.42	0.0016766	227443_at	LURAP1L	2.73	0.00655
ASMM10P052378	**Lyst**	lysosomal trafficking regulator	-8.35	0.0063375	203518_at	LYST	2.21	0.00093
ASMM10P037844	**Klk7**	kallikrein related-peptidase 7 (chymotryptic, stratum corneum)	-8.74	0.000376	239381_at	KLK7	5.24	0.00023
ASMM10P055807	**Gper1**	G protein-coupled estrogen receptor 1	-8.77	0.0012068	210640_s_at	GPER	2.89	8.00E-05
ASMM10P027458	**Kank4**	KN motif and ankyrin repeat domains 4	-8.82	0.0012715	229125_at	KANK4	16.06	0
ASMM10P025449	**Dapp1**	dual adaptor for phosphotyrosine and 3-phosphoinositides 1	-10.02	0.0005713	222859_s_at	DAPP1	2.09	0.00312
ASMM10P026795	**Atp6v0d2**	ATPase, H+ transporting, lysosomal V0 subunit D2	-10.62	0.0060794	1553153_at	ATP6V0D2	2.18	0.02069
ASMM10P025586	**Lrrc7**	leucine rich repeat containing 7	-11.14	0.0002523	233499_at	LRRC7	2.31	0.02334
ASMM10P032656	**Lfng**	LFNG O-fucosylpeptide 3-beta-N-acetylglucosaminyltransferase	-12.33	0.000401	228762_at	LFNG	2.44	0.00201
ASMM10P040613	**Rasd2**	RASD family, member 2	-14.32	0.0028108	223634_at	RASD2	2.3	0.00523
ASMM10P002012	**Pdzk1**	PDZ domain containing 1	-15.77	4.77E-05	205380_at	PDZK1	3.21	0.0207
ASMM10P036964	**Klf7**	Kruppel-like factor 7 (ubiquitous)	-18.91	0.0023523	238517_at	KLF7	2.47	0.00165
ASMM10P015554	**Slc22a3**	solute carrier family 22 (organic cation transporter), member 3	-24.51	0.004432	205421_at	SLC22A3	4.12	2.00E-05
ASMM10P014447	**BC016579**	cDNA sequence, BC016579	-27.15	0.0022393	1559021_at	C3orf52	2.72	0.00756
ASMM10P042115	**Acpp**	acid phosphatase, prostate	-41.13	0.0039846	204393_s_at	ACPP	2.06	0.00135
ASMM10P008650	**Synpo2**	synaptopodin 2	-58.59	4.77E-05	225720_at	SYNPO2	2.09	0.00858
ASMM10P045844	**Map7d2**	MAP7 domain containing 2	-223.84	5.74E-05	228262_at	MAP7D2	3	3.00E-05

Table S2: The top 15 enriched oncogenic signatures in the CRTC1-MAML2-induced murine MEC vs. normal salivary gland tissues are shown.

RANK	Top enriched oncogenic signature	MSigDB gene set	SIZE	ES	NES	FDR q-val
1	RB1/RBL1 loss-induced signature	RB_P107_DN.V1_UP	117	0.53	2.08	0
2	VEGF-induced signature	VEGF_A_UP.V1_DN	161	0.5	2.07	0
3	Late serum response signature	CSR_LATE_UP.V1_UP	134	0.5	2.05	0
4	Oncogenic KRAS induced signature	KRAS.DF.V1_UP	164	0.48	2	0
5	cAMP-induced signature	CAMP_UP.V1_UP	158	0.46	1.89	0.001
6	YAP conserved signature	CORDENONSI_YAP_CONSERVED_SIGNATURE	53	0.53	1.89	0.001
7	Shh-induced signature	GCNP_SHH_UP_LATE.V1_UP	144	0.46	1.88	0.001
8	EIF4G1 regulated signature	SIRNA_EIF4GI_DN	72	0.5	1.84	0.002
9	Down-regulated gene signature during early stages of differentiation of embryoid bodies	ESC_V6.5_UP_EARLY.V1_DN	143	0.44	1.82	0.003
10	Down-regulated gene signature in breast cancer positive for ESR1 vs long-term adapted for estrogen-independent growth	LTE2_UP.V1_DN	149	0.43	1.79	0.003
11	TBK1-induced signature	TBK1.DF_DN	235	0.41	1.77	0.004
12	Oncogenic EGFR-induced signature	EGFR_UP.V1_UP	151	0.43	1.74	0.005
13	ES cell differentiation-inhibited signature	ESC_V6.5_UP_LATE.V1_DN	147	0.42	1.74	0.004
14	SNF5-inhibited signature	SNF5_DN.V1_UP	149	0.42	1.73	0.004
15	ES cell differentiation-inhibited signature	ESC_J1_UP_LATE.V1_DN	146	0.42	1.72	0.005

Table S3: A list of differentially expressed genes in the CRTC1-MAML2-induced murine MEC show overlaps with the CDK4/6-RB integrated signature and/or the RB1/RBL1 loss-induced gene set. *Differential expressed genes in mouse MEC vs normal salivary gland tissues were defined using cut-off criteria of absolute fold change of >=2.0 and FDR p value of =< 0.05. These genes were compared with the CDK4/6-RB integrated signature (Knudsen et al, 2020) and RB_P107_DN.V1_up gene set (Molecular signature database). Red: upregulated; blue: down-regulated.

ProbeName	GeneName	Gene description	Fold Change (Tumor/Normal)	FDR p-value	CDK4/6-RB integrated signature	geneset RB_P107_DN.V1_UP
ASMM10P006922	Birc5	baculoviral IAP repeat-containing 5	138.95	0.0017	BIRC5	
ASMM10P019843	Kif11	kinesin family member 11	66.29	0.0015	KIF11	
ASMM10P036956	Kif22	kinesin family member 22	62.49	0.0019	KIF22	
ASMM10P006949	Ccna2	cyclin A2	61.76	0.0032	CCNA2	
ASMM10P048998	Spag5	sperm associated antigen 5	57.86	0.0008	SPAG5	
ASMM10P006734	Mcm6	minichromosome maintenance deficient 6 (MIS5 homol	56.38	0.0010	MCM6	MCM6
ASMM10P041709	Kif23	kinesin family member 23	45.43	0.0021	KIF23	
ASMM10P008811	Dlgap5	discs, large (Drosophila) homolog-associated protein 5	44.56	0.0012	DLGAP5	
ASMM10P049620	Mcm3	minichromosome maintenance deficient 3 (S. cerevisiae	44.24	0.0011	MCM3	
ASMM10P011830	Cdkn3	cyclin-dependent kinase inhibitor 3	41.12	0.0030	CDKN3	
ASMM10P008087	Ube2c	ubiquitin-conjugating enzyme E2C	41.00	0.0010	UBE2C	
ASMM10P016427	Ndc80	NDC80 homolog, kinetochore complex component (S. c	40.71	0.0016	NDC80	
ASMM10P025023	Exo1	exonuclease 1	39.58	0.0018	EXO1	
ASMM10P011246	Cdca2	cell division cycle associated 2	38.39	0.0017	CDCA2	
ASMM10P000213	Prc1	protein regulator of cytokinesis 1	38.13	0.0022	PRC1	PRC1
ASMM10P019858	Hells	helicase, lymphoid specific	37.94	0.0014	HELLS	
ASMM10P046554	Hmmr	hyaluronan mediated motility receptor (RHAMM)	36.03	0.0034	HMMR	
ASMM10P015707	Ccnf	cyclin F	34.48	0.0023		CCNF
ASMM10P001521	Tk1	thymidine kinase 1	32.95	0.0007	TK1	
ASMM10P041853	Ccnb2	cyclin B2	32.83	0.0020	CCNB2	
ASMM10P050862	Lrr1	leucine rich repeat protein 1	32.61	0.0009	LRR1	
ASMM10P027685	Cdc20	cell division cycle 20	32.02	0.0039	CDC20	
ASMM10P048341	Slit3	slit homolog 3 (Drosophila)	31.97	0.0001		SLIT3
ASMM10P025819	Plk4	polo-like kinase 4	31.34	0.0002	PLK4	
ASMM10P039379	Neil3	nei like 3 (E. coli)	30.85	0.0016	NEIL3	
ASMM10P043142	Ttk	Ttk protein kinase	29.56	0.0010	TTK	
ASMM10P031586	Cenpa	centromere protein A	29.39	0.0009	CENPA	
ASMM10P023597	Nusap1	nucleolar and spindle associated protein 1	28.97	0.0010	NUSAP1	
ASMM10P003012	Tk1	thymidine kinase 1	27.93	0.0006	TK1	
ASMM10P050688	Rrm2	ribonucleotide reductase M2	26.03	0.0013	RRM2	RRM2
ASMM10P052191	Ccnb1	cyclin B1	25.77	0.0031	CCNB1	
ASMM10P009599	Dscc1	defective in sister chromatid cohesion 1 homolog (S. cer	25.68	0.0020	DSCC1	
ASMM10P018536	Lmnb1	lamin B1	25.49	0.0017	LMNB1	
ASMM10P037068	Mki67	antigen identified by monoclonal antibody Ki 67	24.41	0.0056	MKI67	
ASMM10P000899	Fanci	Fanconi anemia, complementation group I	24.35	0.0014	FANCI	
ASMM10P015943	Kifc1	kinesin family member C1	23.52	0.0016	KIFC1	
ASMM10P020242	Mastl	microtubule associated serine/threonine kinase-like	23.43	0.0006	MASTL	
ASMM10P053345	Haus8	4HAUS augmin-like complex, subunit 8	22.99	0.0003	HAUS8	HAUS8
ASMM10P005436	Timeless	timeless circadian clock 1	22.55	0.0009	TIMELESS	
ASMM10P050073	Pole2	polymerase (DNA directed), epsilon 2 (p59 subunit)	22.51	0.0012	POLE2	POLE2
ASMM10P006950	Ccne2	cyclin E2	22.35	0.0022	CCNE2	CCNE2
ASMM10P049219	Col1a1	collagen, type I, alpha 1	21.50	0.0002		COL1A1
ASMM10P029650	Rpa2	replication protein A2	21.23	0.0014		RPA2
ASMM10P037372	Lig1	ligase I, DNA, ATP-dependent	21.03	0.0007	LIG1	LIG1
ASMM10P053525	Ttk	Ttk protein kinase	20.95	0.0017	TTK	
ASMM10P010448	Dhfr	dihydrofolate reductase	20.25	0.0018	DHFR	DHFR
ASMM10P027805	Cdca8	cell division cycle associated 8	19.92	0.0053	CDCA8	
ASMM10P021799	Ube2t	ubiquitin-conjugating enzyme E2T (putative)	19.90	0.0004	UBE2T	
ASMM10P033815	Rad51ap	RAD51 associated protein 1	19.80	0.0014	RAD51AP1	
ASMM10P025273	Gpsm2	G-protein signalling modulator 2 (AGS3-like, C. elegans)	19.73	0.0016	GPSM2	
ASMM10P038436	Rrm1	ribonucleotide reductase M1	19.21	0.0003		RRM1
ASMM10P054508	Ranbp1	RAN binding protein 1	19.04	0.0010	RANBP1	
ASMM10P010564	Depdc1b	DEP domain containing 1B	18.62	0.0031	DEPDC1B	
ASMM10P053814	Rpa1	replication protein A1	17.87	0.0016		RPA1
ASMM10P004473	Mfge8	milk fat globule-EGF factor 8 protein	17.87	0.0057		MFGE8

ASMM10P046014	E2f7	E2F transcription factor 7	17.86	0.0021	E2F7	
ASMM10P023472	Kif18a	kinesin family member 18A	17.75	0.0006	KIF18A	
ASMM10P031648	Tacc3	transforming, acidic coiled-coil containing protein 3	17.70	0.0014	TACC3	
ASMM10P004751	Cenpw	centromere protein W	16.34	0.0031	CENPW	
ASMM10P015787	Dusp1	dual specificity phosphatase 1	16.33	0.0020		DUSP1
ASMM10P031217	Mcm7	minichromosome maintenance deficient 7 (S. cerevisiae	15.72	0.0009	MCM7	MCM7
ASMM10P034073	Col1a2	collagen, type I, alpha 2	15.49	0.0003		COL1A2
ASMM10P008278	Cenpn	centromere protein N	15.05	0.0005	CENPN	
ASMM10P021580	Pcna	proliferating cell nuclear antigen	14.90	0.0004	PCNA	PCNA
ASMM10P049741	Anapc11	anaphase promoting complex subunit 11	14.35	0.0003		ANAPC11
ASMM10P036029	Pold1	polymerase (DNA directed), delta 1, catalytic subunit	14.12	0.0026	POLD1	POLD1
ASMM10P007879	Spc25	SPC25, NDC80 kinetochore complex component, homo	13.54	0.0010	SPC25	
ASMM10P026895	Fbxo5	F-box protein 5	13.45	0.0012	FBXO5	
ASMM10P004448	Oip5	Opa interacting protein 5	13.38	0.0015	OIP5	
ASMM10P035964	Ccne1	cyclin E1	13.10	0.0068		CCNE1
ASMM10P031967	Dck	deoxycytidine kinase	12.87	0.0028	DCK	
ASMM10P015438	Chaf1b	chromatin assembly factor 1, subunit B (p60)	12.82	0.0012	CHAF1B	CHAF1B
ASMM10P040706	Orc6	origin recognition complex, subunit 6	12.26	0.0036	ORC6	ORC6
ASMM10P014139	Mcm4	minichromosome maintenance deficient 4 homolog (S.	11.91	0.0023	MCM4	
ASMM10P052864	Mad2l1	MAD2 mitotic arrest deficient-like 1	11.84	0.0005	MAD2L1	MAD2L1
ASMM10P032535	Rfc2	replication factor C (activator 1) 2	11.78	0.0008	RFC2	RFC2
ASMM10P003125	Rfc3	replication factor C (activator 1) 3	11.20	0.0003	RFC3	RFC3
ASMM10P046297	H2afv	H2A histone family, member V	11.07	0.0005		H2AZ2
ASMM10P007403	Incenp	inner centromere protein	10.99	0.0007	INCENP	INCENP
ASMM10P032431	Kntc1	kinetochore associated 1	10.91	0.0034	KNTC1	
ASMM10P004239	Fancd2	Fanconi anemia, complementation group D2	10.78	0.0006	FANCD2	
ASMM10P034865	Fbln2	fibulin 2	10.77	0.0086		FBLN2
ASMM10P045214	Bgn	biglycan	10.50	0.0028		BGN
ASMM10P047924	Lgals3bp	lectin, galactoside-binding, soluble, 3 binding protein	10.40	0.0011		LGALS3BP
ASMM10P051291	Siva1	SIVA1, apoptosis-inducing factor	10.35	0.0002		SIVA1
ASMM10P013921	Tuba1c	tubulin, alpha 1C	10.26	0.0006	TUBA1C	
ASMM10P055322	S100a4	S100 calcium binding protein A4	10.06	0.0010		S100A4
ASMM10P052751	Cks2	CDC28 protein kinase regulatory subunit 2	9.90	0.0009	CKS2	
ASMM10P053793	Cenpl	centromere protein L	9.70	0.0005	CENPL	
ASMM10P005417	Orc6	origin recognition complex, subunit 6	9.55	0.0066	ORC6	ORC6
ASMM10P009237	Plk4	polo-like kinase 4	9.43	0.0006	PLK4	
ASMM10P004742	Dtymk	deoxythymidylate kinase	9.25	0.0004	DTYMK	
ASMM10P029690	Stmn1	stathmin 1	9.04	0.0032	STMN1	STMN1
ASMM10P013495	Nsmce2	non-SMC element 2 homolog (MMS21, S. cerevisiae)	8.87	0.0002		NSMCE2
ASMM10P002874	Rad51	RAD51 homolog	8.61	0.0024	RAD51	
ASMM10P038038	Trpm1	transient receptor potential cation channel, subfamily M	8.26	0.0004		TRPM1
ASMM10P008626	Kifc5b	kinesin family member C5B	8.22	0.0004	KIFC1	
ASMM10P019495	Cd248	CD248 antigen, endosialin	7.82	0.0055		CD248
ASMM10P008589	Bicc1	bicaudal C homolog 1 (Drosophila)	7.74	0.0009		BICC1
ASMM10P051995	Trip13	thyroid hormone receptor interactor 13	7.73	0.0028	TRIP13	
ASMM10P017150	Nrm	nurim (nuclear envelope membrane protein)	7.54	0.0043		NRM
ASMM10P012579	Atad2	ATPase family, AAA domain containing 2	7.31	0.0012	ATAD2	
ASMM10P006814	Bex1	brain expressed gene 1	7.24	0.0306		BEX2
ASMM10P053240	Coro1a	coronin, actin binding protein 1A	7.12	0.0547	CORO1A	
ASMM10P055129	Fbn1	fibrillin 1	6.85	0.0051		FBN1
ASMM10P030881	Tmem11	transmembrane protein 119	6.74	0.0014		TMEM119
ASMM10P041672	Loxl1	lysyl oxidase-like 1	6.69	0.0067		LOXL1
ASMM10P004194	Vrk1	vaccinia related kinase 1	6.61	0.0028	VRK1	
ASMM10P033486	Mcm2	minichromosome maintenance deficient 2 mitotin (S. ce	6.55	0.0013	MCM2	MCM2
ASMM10P018843	Pola2	polymerase (DNA directed), alpha 2	6.50	0.0032	POLA2	
ASMM10P005234	Siva1	SIVA1, apoptosis-inducing factor	6.30	0.0003		SIVA1
ASMM10P008779	Tssc4	tumor-suppressing subchromosomal transferable fragm	6.24	0.0015		TSSC4
ASMM10P013251	Nup155	nucleoporin 155	6.10	0.0003	NUP155	
ASMM10P014857	Rpl39l	ribosomal protein L39-like	6.08	0.0046	RPL39L	RPL39L
ASMM10P000575	Hexdc	hexosaminidase (glycosyl hydrolase family 20, catalytic	6.04	0.0040		HEXD
ASMM10P000077	Pttg1	pituitary tumor-transforming gene 1	6.03	0.0023	PTTG1	
ASMM10P052540	Uck2	uridine-cytidine kinase 2	5.72	0.0019	UCK2	
ASMM10P049782	Cenpo	centromere protein O	5.71	0.0004	CENPO	
ASMM10P051142	Ubr7	ubiquitin protein ligase E3 component n-recognin 7 (pu	5.67	0.0004		UBR7

ASMM10P039750	Cdh11	cadherin 11	5.62	0.0015		CDH11
ASMM10P044642	Prdx4	peroxiredoxin 4	5.61	0.0014		PRDX4
ASMM10P028831	Smc2	structural maintenance of chromosomes 2	5.49	0.0145	SMC2	
ASMM10P001698	Hmces	5-hydroxymethylcytosine (hmC) binding, ES cell specific	5.49	0.0246		HMCES
ASMM10P016032	Tubb5	tubulin, beta 5 class I	5.44	0.0074	TUBB	
ASMM10P004195	Vrk1	vaccinia related kinase 1	5.18	0.0055	VRK1	
ASMM10P048251	Fancl	Fanconi anemia, complementation group L	5.05	0.0008		FANCL
ASMM10P012117	Cenpf	centromere protein F	4.95	0.0008	CENPF	
ASMM10P031241	Col6a2	collagen, type VI, alpha 2	4.80	0.0059		COL6A2
ASMM10P053968	Prim1	DNA primase, p49 subunit	4.77	0.0010	PRIM1	PRIM1
ASMM10P000539	Timeless	timeless circadian clock 1	4.65	0.0086	TIMELESS	
ASMM10P055423	Hexdc	hexosaminidase (glycosyl hydrolase family 20, catalytic (	4.64	0.0168		HEXD
ASMM10P028755	Melk	maternal embryonic leucine zipper kinase	4.57	0.0012	MELK	
ASMM10P013537	Khdrbs3	KH domain containing, RNA binding, signal transductior	4.50	0.0105		KHDRBS3
ASMM10P037526	Ethe1	ethylmalonic encephalopathy 1	4.39	0.0025		ETHE1
ASMM10P009495	Cenpm	centromere protein M	4.38	0.0007	CENPM	
ASMM10P041291	Anln	anillin, actin binding protein	4.07	0.0045	ANLN	
ASMM10P031270	Col6a1	collagen, type VI, alpha 1	4.07	0.0065		COL6A1
ASMM10P038984	Tssc4	tumor-suppressing subchromosomal transferable fragm	4.04	0.0021		TSSC4
ASMM10P015144	Fstl1	follistatin-like 1	3.92	0.0038		FSTL1
ASMM10P011802	Prim2	DNA primase, p58 subunit	3.91	0.0024	PRIM2	PRIM2
ASMM10P014712	Donson	downstream neighbor of SON	3.86	0.0061	DONSON	
ASMM10P008242	Syce2	synaptonemal complex central element protein 2	3.82	0.0034		SYCE2
ASMM10P051537	Gmnn	geminin	3.82	0.0062	GMNN	GMNN
ASMM10P006739	Mfge8	milk fat globule-EGF factor 8 protein	3.77	0.0481		MFGE8
ASMM10P013494	Nsmce2	non-SMC element 2 homolog (MMS21, S. cerevisiae)	3.63	0.0090		NSMCE2
ASMM10P047817	Dtymk	deoxythymidylate kinase	3.61	0.0029	DTYMK	
ASMM10P035435	Ube2s	ubiquitin-conjugating enzyme E2S	3.58	0.0024	UBE2S	
ASMM10P010728	Dnajc9	DnaJ (Hsp40) homolog, subfamily C, member 9	3.57	0.0021	DNAJC9	DNAJC9
ASMM10P007750	Nuf2	NUF2, NDC80 kinetochore complex component, homol	3.53	0.0106	NUF2	
ASMM10P006786	Pml	promyelocytic leukemia	3.52	0.0651		PML
ASMM10P001795	Fancl	Fanconi anemia, complementation group L	3.40	0.0055		FANCL
ASMM10P053818	Serpinf1	serine (or cysteine) peptidase inhibitor, clade F, member	3.38	0.0149		SERPINF1
ASMM10P018936	Fen1	flap structure specific endonuclease 1	3.36	0.0064	FEN1	FEN1
ASMM10P015507	Dynlt1a	dynein light chain Tctex-type 1A	3.21	0.0385		DYNLT1
ASMM10P022399	Pdss1	prenyl (solanesyl) diphosphate synthase, subunit 1	3.20	0.0017	PDSS1	
ASMM10P041390	Zwint	ZW10 interactor	3.20	0.0068	ZWINT	
ASMM10P002597	Rfc4	replication factor C (activator 1) 4	3.19	0.0050	RFC4	
ASMM10P040449	Hmgb2	high mobility group box 2	3.16	0.0039	HMGB2	
ASMM10P000235	Pcolce	procollagen C-endopeptidase enhancer protein	3.11	0.0047		PCOLCE
ASMM10P040819	Gins3	GINS complex subunit 3 (Psf3 homolog)	3.01	0.0254	GINS3	
ASMM10P030961	Rfc5	replication factor C (activator 1) 5	2.91	0.0125		RFC5
ASMM10P033794	Ncapd2	non-SMC condensin I complex, subunit D2	2.89	0.0211	NCAPD2	NCAPD2
ASMM10P051187	Vrk1	vaccinia related kinase 1	2.81	0.0433	VRK1	
ASMM10P016597	Dynlt1b	dynein light chain Tctex-type 1B	2.77	0.0529		DYNLT1
ASMM10P047290	Prr11	proline rich 11	2.75	0.0214	PRR11	
ASMM10P030288	Slbp	stem-loop binding protein	2.53	0.0084	SLBP	SLBP
ASMM10P039176	Gins4	GINS complex subunit 4 (Sld5 homolog)	2.50	0.0043		GINS4
ASMM10P030711	Cdk1	cyclin-dependent kinase 1	2.50	0.0169	CDK1	
ASMM10P005342	Fkbp10	FK506 binding protein 10	2.39	0.0230		FKBP10
ASMM10P006957	Cdc25c	cell division cycle 25C	2.38	0.0233	CDC25C	
ASMM10P005642	Syce2	synaptonemal complex central element protein 2	2.32	0.0723		SYCE2
ASMM10P032837	Rpa3	replication protein A3	2.20	0.0403	RPA3	RPA3
ASMM10P054014	Pttg1	pituitary tumor-transforming gene 1	2.18	0.0123	PTTG1	
ASMM10P052843	Ezh2	enhancer of zeste homolog 2 (Drosophila)	2.16	0.0077	EZH2	
ASMM10P006425	Fen1	flap structure specific endonuclease 1	2.16	0.0179	FEN1	FEN1
ASMM10P039609	Rnaseh2	ribonuclease H2, large subunit	2.08	0.0760	RNASEH2A	
ASMM10P030243	Tyms	thymidylate synthase	2.02	0.0466	TYMS	
ASMM10P017089	Nelfe	negative elongation factor complex member E, Rdbp	-2.08	0.0093		NELFE
ASMM10P012900	Cenpm	centromere protein M	-2.13	0.0318	CENPM	
ASMM10P021470	Ncaph	non-SMC condensin I complex, subunit H	-2.21	0.0326		NCAPH
ASMM10P055542	Lck	lymphocyte protein tyrosine kinase	-2.21	0.0241		LCK
ASMM10P018890	Naa40	N(alpha)-acetyltransferase 40, NatD catalytic subunit, hc	-2.24	0.0265		NAA40
ASMM10P041093	Spire2	spire homolog 2 (Drosophila)	-2.40	0.0120		SPIRE2

ASMM10P029122	Tek	endothelial-specific receptor tyrosine kinase	-2.40	0.0236		TEK
ASMM10P027997	Hmgn2	high mobility group nucleosomal binding domain 2	-2.41	0.0060	HMGN2	HMGN2
ASMM10P024150	Fam83d	family with sequence similarity 83, member D	-2.60	0.0055	FAM83D	
ASMM10P020718	Spc25	SPC25, NDC80 kinetochore complex component, homo	-2.93	0.0059	SPC25	
ASMM10P007419	Cdk2	cyclin-dependent kinase 2	-3.29	0.0334		CDK2
ASMM10P025644	Car2	carbonic anhydrase 2	-3.33	0.0304		CA2
ASMM10P001705	Cdc25b	cell division cycle 25B	-3.45	0.0009	CDC25B	CDC25B
ASMM10P002596	Lrp8	low density lipoprotein receptor-related protein 8, apol	-3.59	0.0075	LRP8	
ASMM10P011088	Efs	embryonal Fyn-associated substrate	-3.68	0.0017		EFS
ASMM10P000792	Srpx2	sushi-repeat-containing protein, X-linked 2	-3.75	0.0026		SRPX2
ASMM10P027625	Rad54l	RAD54 like (S. cerevisiae)	-3.83	0.0036	RAD54L	
ASMM10P049384	Tubg1	tubulin, gamma 1	-4.03	0.0035		TUBG1
ASMM10P052188	Cenph	centromere protein H	-4.07	0.0007		CENPH
ASMM10P014846	Abat	4-aminobutyrate aminotransferase	-4.12	0.0043		ABAT
ASMM10P055512	Kif2c	kinesin family member 2C	-4.39	0.0010	KIF2C	
ASMM10P033393	Actg2	actin, gamma 2, smooth muscle, enteric	-4.51	0.0268		ACTG2
ASMM10P029154	Usp1	ubiquitin specific peptidase 1	-4.68	0.0046	USP1	
ASMM10P018882	Vegfb	vascular endothelial growth factor B	-5.80	0.0002		VEGFB
ASMM10P001611	Pif1	PIF1 5'-to-3' DNA helicase homolog (S. cerevisiae)	-8.07	0.0713	PIF1	
ASMM10P055361	Gstm7	glutathione S-transferase, mu 7	-8.39	0.0006		GSTM2
ASMM10P033810	Kcna1	potassium voltage-gated channel, shaker-related subfam	-9.07	0.0015		KCNA1
ASMM10P029720	E2f2	E2F transcription factor 2	-9.16	0.0004	E2F2	
ASMM10P005321	Fignl1	fidgetin-like 1	-11.33	0.0002	FIGNL1	
ASMM10P041374	Chek1	checkpoint kinase 1	-11.74	0.0004	CHEK1	
ASMM10P010539	Cenpk	centromere protein K	-14.01	0.0001	CENPK	CENPK
ASMM10P012899	Cenpm	centromere protein M	-14.80	0.0001	CENPM	
ASMM10P038765	Hirip3	HIRA interacting protein 3	-30.29	0.0002	HIRIP3	
ASMM10P006959	Cdc45	cell division cycle 45	-162.17	0.0001	CDC45	CDC45

Table S4: Primer sequences used for the qRT-PCR analysis in this study. *The primer sequences were presented in the 5' to 3' direction.

Genes	Forward primer	Reverse primer	Amplicon (bp)
CRTC1-MAML2	TTCGAGGAGGTCATGAAGGA	TTGCTGTTGGCAGGAGATAG	101
LINC00473	AAACGCGAACGTGAGCCCCG	CGCCATGCTCTGGCGCAGTT	134
GAPDH	CAATGACCCCTTCATTGACC	GACAAGCTTCCCGTTCTCAG	106
Areg	GGTCTTAGGCTCAGGCCATT	TGGTCCCCAGAAAGCGATTC	84
Nr4a2	GAGCTGGGCTGCTCGAC	AAGGCATGGCTTCAGCAGAG	197
Mcm6	TCGTCTCACACACTACGATCAC	GGCTAAGGGTAGGACAGCAC	185
Prc1	GGAGCAAGAACTCCGTGACA	TCACGCCTCGATTCCTTTG	135
Rrm2	TGGAGCTGGGTTTTAACAAGATTT	TCAGCGAAGAACACACGATCA	199
Haus8	AAAAAGGTTTCTGGAGCGGC	TCACTTGGCTCTCTTCTCTGC	91
Pole2	CAAGTATCCACCAAAGTAGCCGTT	ACCCTTTGTCTGAATTCCTGTGT	121
Ccne2	ATTTGGCTTTGCTGAATGAAGT	TCCAGGTTGTTAACTTCAGTGTT	143
Lig1	CCCACAAAGTCCCCTGAGTC	AACTGCTCAGTGTCTTGGCA	136
Dhfr	TAAACTTCTCCCAGAATACCCAG	GAGGGGAGCAGAGAACTTGAAA	127
Mcm7	ACAGATGGCACAGGGCTTAC	AAGCGGCCAGCTTCTCATAG	158
Pcna	TGAACCTCACCAGCATGTCC	TCTGGGATTCCAAGTTGCTCC	181
Hprt	CTGGTGAAAAGGACCTCTCGAAG	CCAGTTTCACTAATGACACAAACG	141
